# Tyrosine-capped gold nanoparticles enable cysteine-free peptide loading, enhancing the antipseudomonal efficacy of scorpion-derived AamAP1-Lys-NH_2_ in a burn wound infection model

**DOI:** 10.1039/d6tb00168h

**Published:** 2026-06-22

**Authors:** Rosalind J. Van Wyk, Mandelie van der Walt, June C. Serem, A. James Mason, Megan J. Bester, Anabella R. M. Gaspar

**Affiliations:** a Department of Biochemistry, Genetics and Microbiology, Faculty of Natural and Agricultural Sciences, University of Pretoria 0002 South Africa anabella.gaspar@up.ac.za; b Department of Anatomy, Faculty of Health Sciences, University of Pretoria 0002 South Africa; c Institute of Pharmaceutical Science, School of Cancer & Pharmaceutical Sciences, King's College London SE1 9NH UK james.mason@kcl.ac.uk

## Abstract

Cytotoxicity and instability in complex physiological environments remain key barriers to the clinical translation of antimicrobial peptides (AMPs) especially for infected wounds, which contain protease-rich exudate and are highly sensitive to toxicity that can impede healing. This is despite the strong potential of AMPs to combat antimicrobial resistance, with efficacy against a broad spectrum of pathogens including multidrug-resistant *Pseudomonas aeruginosa*. Loading AMPs onto gold nanoparticles (AuNPs) offers a promising strategy to protect against degradation, control release, and reduce cytotoxicity, however conventional conjugation methods rely on cysteine residues or linker molecules and typically achieve low peptide loading. Here, we present a robust method for loading the AMP, AamAP1-Lys-NH_2_, onto tyrosine capped gold nanoparticles (Tyr-AuNPs) that achieves nearly complete peptide loading without the need for cysteine or linker residues, while simultaneously reducing cytotoxicity and conferring resistance to trypsin degradation. Fourier-transform infrared (FTIR) spectroscopy confirmed distinct surface chemistry between Tyr-AuNPs and AMP-coated AuNPs, while confocal microscopy verified peptide adsorption. Cumulative release studies showed gradual aggregate dissociation with slow AMP release in water (20% after 1 hour), and faster, complete release in PBS (100% after 1 hour), demonstrating potential tuneable delivery under physiological conditions. *In vivo*, both AamAP1-Lys-NH_2_-AuNPs and its all-d-enantiomer significantly improved survival of *Galleria mellonella* larvae with *P. aeruginosa* burn wound infections (80–85% survival), compared to 55% with free AMP. Since both enantiomers performed equally well, the additional costs of d-enantiomer synthesis are unwarranted when using AuNP delivery. Overall, this study identifies AamAP1-Lys-NH_2_-AuNPs as a promising therapeutic candidate for the topical treatment of infected wounds, with the Tyr-AuNP platform enabling efficient peptide loading while preserving antimicrobial activity, enhancing stability and minimising cytotoxicity.

## Abstract 2

Tyrosine-capped AuNPs enable near-complete loading of AamAP1-Lys-NH_2_, reduce cytotoxicity and protease degradation and preserve antimicrobial activity. Nanoparticles improve survival in *P. aeruginosa* burn wound infections, supporting wound therapy.

## Graphical abstract

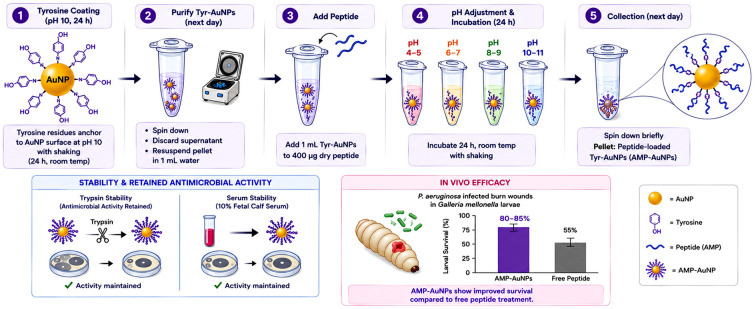

## Introduction

Burn wounds often lead to serious complications, with secondary bacterial infections being a major contributor to delayed healing and increased morbidity.^1^ Among the most problematic pathogens in this context is *Pseudomonas aeruginosa*, an opportunistic Gram-negative bacterium known for its ability to resist different antibiotic classes, colonise damaged tissue, evade host immune responses, and form biofilms.^2,3^ The high prevalence of multidrug-resistant (MDR) *P. aeruginosa* strains in clinical settings makes treatment particularly challenging, especially in burn patients where the skin barrier is compromised.^4,5^ These MDR strains are also a major cause of persistent skin and soft tissue infections (SSTIs), such as gangrenous cellulitis and necrotising fasciitis, which are notoriously difficult to treat and can become life-threatening.^3^ Given these challenges, and the limited efficacy of existing treatments, there is an urgent need to develop alternative therapeutics to combat MDR *P. aeruginosa* infections.

Antimicrobial peptides (AMPs), structurally diverse components of the innate immune systems of various organisms, have emerged as promising candidates for the treatment of infections caused by antimicrobial resistant (AMR) bacteria.^6^ These peptides typically exert their effects through non-specific, multifaceted membranolytic mechanisms, offering broad-spectrum activity against various pathogens and a low likelihood of inducing resistance.^6^ Despite their potent *in vitro* activity, AMPs often fail to demonstrate comparable efficacy in more complex *in vivo* environments.^7^ This is largely due to drawbacks associated with AMP susceptibility to proteolytic degradation, binding to serum proteins, short circulating half-life and cytotoxicity.^8^ These challenges are particularly critical in the context of burn or chronic wound infections, where the local physiological conditions can significantly impair AMP stability and bioavailability.^9^ Therefore, overcoming these barriers is essential for the successful development of AMP-based therapeutics for clinical application.

A common strategy to enhance the proteolytic stability of AMPs is the substitution of l-amino acids with d-amino acids or other non-proteogenic residues. These modifications can increase resistance to enzymatic degradation and, in many cases, improve antimicrobial activity compared to their unmodified counterparts.^10,11^ However, the extended residence time of such modified AMPs in biological systems may also lead to increased cytotoxicity.^11^

Alternatively, a plethora of different drug delivery systems exist which can be used to improve proteolytic stability, potency and in many cases reduce toxicity of AMPs.^12^ In particular, gold nanoparticles (AuNPs) have been extensively researched for this purpose and have unique and versatile properties compared with other biomedical delivery systems.^13^ Some of the advantages of AuNPs are the ease of synthesis through various methods that enable control over size, shape, and morphology, a high surface-area-to-volume ratio, and adaptable surface chemistry.^14^ They also exhibit low toxicity and good biocompatibility, making AuNPs suitable for a wide range of biomedical applications.^15^ Many biomolecules, such as antibiotics, antibodies, proteins, enzymes, aptamers, peptides and amino acids can be used to functionalise the AuNP surface, generally through electrostatic interaction, hydrogen bonding or direct conjugation *via* thiol or amine groups.^16,57^

Specifically, the field of AuNPs loaded with functional AMPs (AMP-AuNPs) is a novel emerging approach that holds potential for translation into the clinic.^17,18^ Currently, no AMP-AuNP antibacterial therapies have entered clinical trials yet, with only AuNPs loaded with proinsulin, C19-A3 AuNP, in phase I trials for the treatment of type I diabetes.^19^ Nevertheless, several AMP-AuNP systems have demonstrated significant antibacterial properties in preclinical studies and show promise for future development.^18,20,21^

A key limitation in this field is that successful peptide functionalisation of AuNPs typically relies on the presence of specific amino acid residues with affinity for gold. Among these, cysteine is most widely used due to the formation of strong covalent gold-thiol (Au-S) bonds.^54,55^ Peptides lacking cysteine in their native sequence are often chemically modified or extended with cysteine or thiolated linkers to enable attachment to the nanoparticle surface. In other cases, direct peptide adsorption may depend on residues such as tryptophan, lysine, or arginine, which interact with AuNPs through weaker π-metal or electrostatic interactions.^54,55^ These interactions can result in poor surface coverage, variable loading efficiency, and limited reproducibility.

To address these challenges, the present work introduces a tyrosine-capped AuNP (Tyr-AuNP) platform that enables efficient, pH-dependent adsorption of an AMP without altering its native amino acid sequence or incorporating cysteine or other thiol-based linkers. In this system, tyrosine capping plays a critical stabilising role, maintaining the morphology and surface chemistry of the AuNPs such that electrostatic or π-interactions with peptides can occur efficiently.

Although amino acid- or peptide-capped AuNPs, including tyrosine-based systems, have been previously reported, they often incorporate cysteine residues to ensure nanoparticle stability and peptide attachment.^21,54,55^ Other amino acids such as tryptophan have also been explored as capping agents for AuNP synthesis, with reported differences in nanoparticle size, stability, and cytotoxicity depending on the amino acid used.^58^ However, these systems have not typically been investigated in the context of subsequent peptide loading. In contrast, our study demonstrates that Tyr-AuNPs can achieve complete peptide loading even in the absence of cysteine, with tyrosine capping providing sufficient surface stabilisation to support pH-mediated adsorption of unmodified AMPs. This strategy yields high loading efficiency while preserving the peptide's native sequence and biological activity.

To illustrate this approach, we focused on advancing the therapeutic potential of AamAP1-Lys-NH_2_, a broad-spectrum AMP derived from the venom of the African *Androctonus amoreuxi* scorpion. In our previous work, AamAP1-Lys-NH_2_ demonstrated the most potent activity among a panel of 46 peptides against a wide range of ESKAPE pathogens.^22^ Its unamidated precursor, AamAP1-Lys, was initially derived from the parent peptide AamAP1 and exhibited an α-helical structure and promising antimicrobial potential.^23^ However, upon amidation the resulting peptide AamAP1-Lys-NH_2_ showed increased structural flexibility which resulted in improved penetration of Gram-negative membranes, increased membrane permeabilisation and faster bactericidal activity.^23^ Furthermore, the peptide conferred improved protection against *A. baumannii* ATCC 17978 infection in an *in vivo Galleria mellonella* burn wound model, demonstrating its therapeutic potential.

To further develop AamAP1-Lys-NH_2_ as a viable therapeutic candidate, this study aimed to enhance its stability by substituting all l-amino acids with their d-enantiomers, and to reduce potential toxicity by incorporating both the l- and d-forms into a nano-gold delivery system.

## Materials and methods

### Peptides

Crude samples of AamAP1-Lys-NH_2_ (FLFKLIPKAIKKLISKFK-NH_2_) were obtained from Biosynth® (Cleveland, UK) and purified using preparative reverse-phase high performance liquid chromatography (RP-HPLC) to >95% purity. The peptide was lyophilised, weighed, and stored in low binding Eppendorf tubes until used. The all d-amino acid analogue, d-AamAP1-Lys-NH_2_ (d-FLFKLIPKAIKKLISKFK-NH_2_; all l-amino acids replaced with d-amino acids, l-Ile residues replaced with d-Allo-Ile, >95% purity), was obtained from GenScript (Piscataway NJ, USA).

### Synthesis and characterisation of AuNPs

**Fig. 1:**
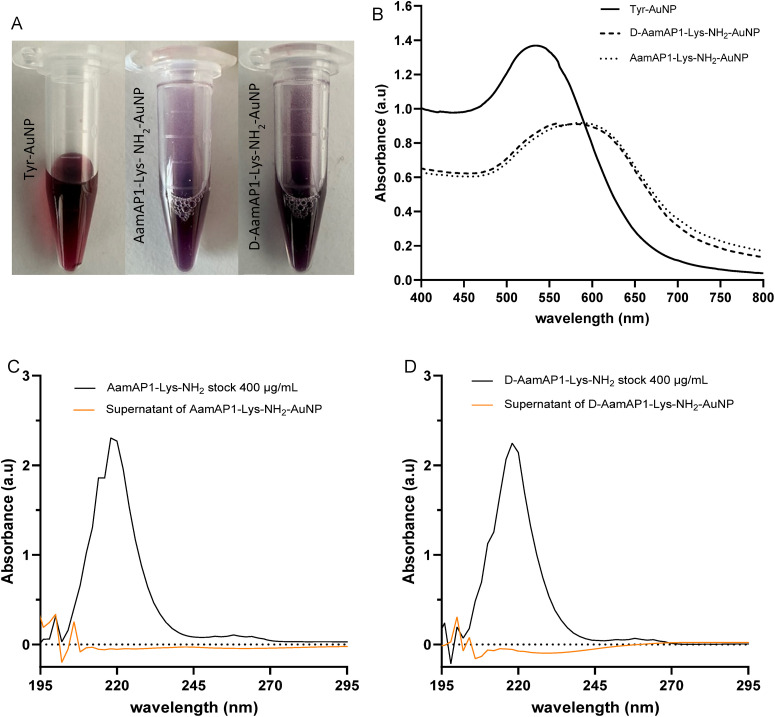
Synthesis of Tyr- and AMP-AuNPs at pH 10.0 and their characterisation with UV-vis spectroscopy. (A) Visible colour change of AuNP products before and after the addition of AMPs from ruby red (Tyr-AuNP) to blue/purple (AamAP1-Lys-NH_2_-AuNP and d-AamAP1-Lys-NH_2_-AuNP) at pH 10.0. (B) Representative UV-vis spectra of Tyr-AuNP, AamAP1-Lys-NH_2_-AuNP and d-AamAP1-Lys-NH_2_-AuNP (100 µg mL^−1^) showing the SPR band shift after addition to AMPs at pH 10.0. (C) Representative UV-vis spectra of the AamAP1-Lys-NH_2_ stock solution (400 µg mL^−1^) and the AamAP1-Lys-NH_2_-AuNP supernatant, showing absence of peptide in the supernatant, suggesting successful loading onto AuNPs at pH 10.0 and precipitation in the pellet. (D) Representative UV-vis spectra of the d-AamAP1-Lys-NH_2_ stock solution (400 µg mL^−1^) and the d-AamAP1-Lys-NH_2_-AuNP supernatant, showing absence of peptide in the supernatant, suggesting successful loading onto AuNPs at pH 10.0 and precipitation in the pellet.

AuNPs were synthesised fresh for each experiment using a modified version of the chemical synthesis method described by Jia and co-workers.^21^ First, Tyr-AuNPs were prepared by adding 0.1 M sodium hydroxide (NaOH) to 750 µL of 10 mM l-tyrosine (reducing and capping agent) in distilled deionised water (ddH_2_O) to adjust the pH to between 11 and 12. Thereafter, 250 µL of 10 mM tetrachloroauric acid (H[AuCl_4_]) was added to the l-Tyr solution dropwise, while gently mixing. This pH is crucial to ensure the hydroxyl group of Tyr is deprotonated, resulting in the reduction of Au^3+^ to Au^0^ to form the Tyr capped AuNPs. Afterwards, the solution was left at room temperature for 24 h in the dark on a shaker (Pelco; Fresno, California, USA) set at 50 rpm. A change in the solution colour from light yellow to ruby red indicated the successful synthesis of Tyr-AuNPs (Fig. 1A).

To load the AMPs onto the AuNPs, the Tyr-AuNPs were collected by centrifugation for 25 min at 18 000 × *g* and the supernatants carefully removed and discarded. Each Tyr-AuNP pellet was resuspended in 976 µL sterile ddH_2_O and transferred to 400 µg dry weight of AamAP1-Lys-NH_2_ or d-AamAP1-Lys-NH_2_. Immediately thereafter, 0.1 M NaOH was added to each suspension to reach a pH of 10.0, followed by the addition of 20 µL of the stabilising agent, Poloxamer 188 (7 mM) to reach a final volume of 1 mL. A Tyr-AuNP control was also prepared by resuspending a Tyr-AuNP pellet in 976 µL sterile ddH_2_O, followed by the addition of 4 µL 0.1 M NaOH and 20 µL Poloxamer 188. The final peptide concentration of each nano-gold reaction mixture was 400 µg mL^−1^. The suspensions were then kept in the dark on a shaker at 50 rpm for 24 h to allow the peptides to bind to the Tyr-capped AuNPs, as indicated by a visible colour change to dark blue or purple (Fig. 1A). The AMP-AuNPs (AamAP1-Lys-NH_2_-AuNP and d-AamAP1-Lys-NH_2_-AuNP) and Tyr-AuNP control were collected by centrifugation at 18 000 × *g* for 1 min in the case of the peptides and 25 min for the control. The damp pellets and supernatants were collected and stored separately for further analysis.

### Surface plasmon resonance band analysis

To confirm the successful formation of AMP-AuNPs and Tyr-AuNPs, UV-visible (UV-vis) spectroscopy was performed to assess their characteristic surface plasmon resonance (SPR) bands. Following centrifugation, the AMP-AuNP and Tyr-AuNP pellets were resuspended in sterile ddH_2_O to a concentration of 400 µg mL^−1^, then diluted 4-fold to a final peptide concentration of 100 µg mL^−1^. UV-Vis spectra of the diluted pellets were recorded over the 400–800 nm range on two separate occasions using a UV-vis spectrophotometer (Optizen POP).

### AMP loading efficiency

To determine the peptide loading efficiency of the AMP-AuNPs, UV-vis absorbance spectra (190–700 nm) were obtained for 400 µg mL^−1^ free AMP stock solutions in sterile ddH_2_O, as well as for the supernatants of AMP-AuNPs and the Tyr-AuNP control. The absorbance spectrum of the Tyr-AuNP control supernatant was used as the blank and subtracted from the spectra of the AMP-AuNP supernatants. The absorbance peak at wavelength ∼215 nm, where peptide backbone absorbs the maximum light, was subsequently used to calculate the percentage bound AMP using the following equation:
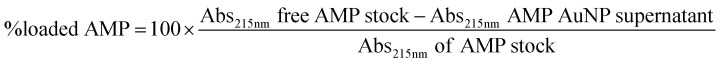


### Morphology and size analysis of AMP-AuNPs

The morphology and sizes of the Tyr-AuNPs and AMP-AuNPs were analysed using transmission electron microscopy (TEM). A JEM-2100F TEM (JEOL, Tokyo, Japan), operating at an accelerating voltage of 115 kV, was used to acquire images of the AuNPs. The Tyr-AuNP and AMP-AuNP pellets were washed three times with sterile ddH_2_O, then resuspended to a concentration of 400 µg mL^−1^. The suspension was divided equally into four tubes, each containing 100 µg of peptide. The samples were centrifuged at 18 000 × *g* for 2 min, and the resulting pellets were resuspended in 10 µL of sterile ddH_2_O to achieve a final peptide concentration of 10 mg mL^−1^. A 20 µL sample of resuspended AuNP pellet (10 mg mL^−1^ peptide) in ddH_2_O was applied to carbon-coated copper grids and allowed to dry overnight in a desiccator. The size distribution was determined by measuring the diameter of one hundred Tyr-AuNPs and AMP-AuNPs in each sample, on two different occasions (*n* = 200) using ImageJ v1.6 software (National Institutes of Health, Maryland, USA).

### Attenuated total reflectance – Fourier transform infrared spectroscopy

To determine the functional groups that are present on the surface of the Tyr-AuNP and AMP-AuNPs, as well as those present in free AamAP1-Lys-NH_2_ and d-AamAP1-Lys-NH_2_, attenuated total reflectance-Fourier transform infrared (ATR-FTIR) spectroscopy was employed. The Tyr-AuNP and AMP-AuNPs pellets were washed three times with sterile ddH_2_O then resuspended to a concentration of 400 µg mL^−1^. The suspension was divided equally into four tubes, each containing 100 µg of peptide. The samples were centrifuged at 18 000 × *g* for 2 min, and the resulting pellets were resuspended in 10 µL of sterile ddH_2_O to achieve a final AMP-AuNP concentration of 10 mg mL^−1^. The ATR-FTIR spectra for the AMP-AuNPs and the free AamAP1-Lys-NH_2_ and d-AamAP1-Lys-NH_2_ (10 mg mL^−1^) were recorded on a Jasco FT/IR-4× instrument fitted with a triglycine sulphate (TGS) detector at a resolution of 4 cm^−1^.

### Confocal fluorescence microscopy

The intrinsic fluorescence of amino acids (excitation wavelength of 520 nm) was exploited to visualise Tyr and AMPs on the surface of the AuNPs using confocal fluorescence microscopy *via* the adapted method described by Verma *et al.*^24^ The AMP-AuNP solutions made up to a final concentration of 10 mg mL^−1^ as described above, were transferred to clean glass slides, covered with a glass cover slip and fastened with Sellotape. The samples were analysed on a Zeiss LSM confocal microscope on a Fast 880 Airyscan mode. A 63 × 0.9 NA objective was used, and an excitation wavelength of 514 nm was selected. The fluorescence emission was recorded on a detector after passing through a 645 nm filter. The images were processed using ZEN Black 2.3 SPI.

### *In vitro* AMP release studies at physiological pH

The release of AamAP1-Lys-NH_2_ and d-AamAP1-Lys-NH_2_ from the AMP-AuNPs was measured over 60 min in PBS (0.01 M, pH 7.4) and ddH_2_O (pH 7.0). The AMP-AuNPs pellets (400 µg) were resuspended in PBS or ddH_2_O to a final AMP concentration of 200 µg mL^−1^ and incubated at room temperature in the dark. The supernatants were collected at time points, 10, 30 and 60 min, by centrifugation at 18 000 × *g* for 2 min. The UV-vis spectra of the supernatants and the free AMPs (200 µg mL^−1^) were recorded in the spectral range of 200–250 nm. The blank was PBS or ddH_2_O. Duplicate experiments were performed and the percentage cumulative release of AMPs from the AMP-AuNPs was determined using the absorbance of the peptide backbone at a wavelength of 215 nm and the following equation:
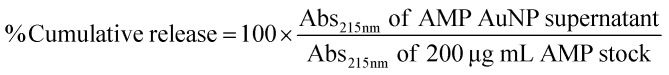


### Aggregation of AMP-AuNPs

To evaluate the effect of pH on peptide loading onto AuNPs and the resulting nanoparticle aggregation, prepared Tyr-capped AuNP pellets were resuspended in 976 µL sterile ddH_2_O and combined with 400 µg (dry weight equivalent) of peptide. The pH of each suspension was subsequently adjusted using 0.1 M NaOH to achieve final pH ranges of 4–5, 6–7, 8–9, or 10–11. Thereafter, 20 µL of Poloxamer 188 (7 mM) was added as a stabilising agent to obtain final reaction volumes of 1 mL. The final peptide concentration in each AMP-AuNP formulation was 400 µg mL^−1^. The suspensions were incubated for 24 h at room temperature in the dark with gentle shaking (50 rpm) to facilitate peptide adsorption onto the Tyr-capped AuNPs. Peptide loading and nanoparticle aggregation were visually indicated by a colour change in the suspensions (Fig. S3). Peptide loading efficiency was determined as mentioned before. Aggregate formation was assessed using an Olympus DP23 stereomicroscope. Briefly, 100 µL of each AMP-AuNP suspension was transferred to individual wells of a 96-well plate in duplicate and imaged at magnifications of 1.6×, 2.5×, and 4×.

### Dissociation of AMP-AuNPs aggregates

To evaluate the dissociation of AMP-AuNP aggregates under neutral pH conditions and in the presence or absence of salts, AMP-AuNP pellets formed overnight at pH 10.0 were resuspended in 1 mL sterile ddH_2_O (pH 7.0) or PBS (pH 7.4). Aggregate dissociation over time was qualitatively assessed using an Olympus DP23 stereomicroscope. Briefly, 100 µL of each AMP-AuNP suspension was transferred to individual wells of a 96-well plate and imaged at magnifications of 1.6×, 2.5×, and 4× immediately following resuspension (0 min) and after incubation for 10, 30, and 60 min. Representative images were captured at each time point to qualitatively compare aggregate dissociation in neutral aqueous conditions (ddH_2_O) and salt-containing conditions (PBS).

### Determination of minimum inhibitory and bactericidal concentrations

*P. aeruginosa* PAO1 was obtained from the American Type Culture Collection (ATCC). Briefly, bacteria were streaked out onto tryptic soy broth (TSB) (Merck, Darmstadt, Germany) agar plates and incubated overnight at 37 °C. The next day, 3–5 colonies were picked and resuspended in 4 mL Mueller Hinton broth (MHB) to reach an optical density at 600 nm (OD_600nm_) of 0.1. The bacterial inoculum was further diluted 100× to an OD_600nm_ of 0.001 to yield approximately 2 × 10^6^ CFU mL^−1^. Free AMP and AMP-AuNP solutions of 5 mg mL^−1^ were prepared in sterile ddH_2_O. The AMPs and AMP-AuNPs were two-fold serially diluted in MHB in polypropylene 96-well plates (Thermo Fisher Scientific, Roskilde, Denmark). Following dilution, 50 µL of *P. aeruginosa* PAO1 suspension (1 × 10^6^ CFU per mL^−1^) was added to 50 µL of each AMP dilution, yielding a total volume of 100 µL per well and a final AMP concentration range of 0.125–64 µg mL^−1^. Sterility controls contained MHB only while the positive control was 128 µg mL^−1^ polymyxin B (Merck, Darmstadt, Germany) and the negative controls included cells treated with ddH_2_O or Tyr-AuNPs. The Tyr-AuNP control was equivalent to the highest concentration of AMP-AuNPs. The plate was incubated for 24 h at 37 °C. The minimum inhibitory concentration (MIC) was visually determined as the lowest concentration showing no visible turbidity, indicating inhibition of bacterial growth. The minimal bactericidal concentration (MBC) was determined by spread plating 100 µL from the wells at the MIC and above onto TSB agar plates. The MBC was defined as the concentration at which no colonies were observed on the agar plate after 16 h stationary incubation at 37 °C. Inhibition activity was determined as an average across three biological repeats, each performed in triplicate.

### Serum and protease stability of AMP-AuNPs

Free AMPs and AMP-AuNPs were resuspended in either 10% fetal calf serum (FCS) or trypsin (2.5 or 5 mM; 200 FIP-U per g in ddH_2_O) and incubated at 37 °C for 60 min. Enzymatic activity was subsequently halted by heating the samples at 60 °C for 20 min. Serial dilutions of the pre-treated AMPs and AMP-AuNPs were prepared in a 96-well polypropylene plate as described for the MIC assay. Subsequently, 50 µL of *P. aeruginosa* PAO1 bacterial suspension was added to each well to obtain a final bacterial cell density of 1 × 10^6^ CFU per mL^−1^ and AMP or AMP-AuNP concentration range of 0.125 to 64 µg mL^−1^. Sterility controls contained MHB only while the positive control was 128 µg mL^−1^ polymyxin B (Merck, Darmstadt, Germany) and the negative controls included cells treated with ddH_2_O or Tyr-AuNPs. The plate was incubated for 24 h at 37 °C and the MIC and MBC determined as described for the MIC assay. Serum and protease stability was determined as an average across three biological repeats, each performed in triplicate.

### Cytotoxicity

The free AMPs and AMP-AuNPs were screened for cytotoxicity against human keratinocytes (HaCat cells) purchased from ECACC. HaCat cells (90 µL) were cultured in Dulbecco's modified Eagles medium (DMEM) supplemented with 10% FCS and 1% antibiotic–antimycotic and plated at 1.1 × 10^5^ cells per mL in a NEST 96-well microtiter plate (Whitehead Scientific, Modderfontein, Gauteng, SA). Following an overnight incubation at 37 °C, 5% CO_2_, and 95% humidity, 10 µL of each peptide concentration was added to the cells to reach final AMP and AMP-AuNP concentrations of 16 to 64 µg mL^−1^. The positive control consisted of HaCat cells treated with 10 µL of 0.1% Triton-X100, while negative controls included HaCat cells treated with PBS or Tyr-AuNPs. The plates were then incubated for 21 h at 37 °C, 5% CO_2_ and 90% humidity, before adding 11 µL of 3-(4,5-dimethylthiazol-2-yl)-2,5-diphenyltetrazolium bromide (MTT) reagent (1 mg mL^−1^ in 1 M PBS) to each well. After a further 3 h incubation, the media was removed, the plates blot dried, and the formazan crystals solubilised with 50 µL of a 25% (v/v) dimethyl sulfoxide (DMSO) in ethanol solution. The absorbance was measured at 570 nm with a FLUOstar® Omega spectrophotometer (BMG Labtech, Germany) and percentage cell death determined relative to the negative controls.

### *In vivo Galleria mellonella* burn wound infection model

*G. mellonella* larvae were obtained from and maintained by the Forestry and Agricultural Biotechnology Institute (FABI) Biocontrol center at the University of Pretoria, South Africa. A similar procedure as used by van der Walt *et al.*^25^ and Di Blasio *et al.*,^26^ and first introduced by Maslova *et al.*,^56^ was followed. Healthy, final instar *G. mellonella* larvae of similar weight (200 ± 20 mg) were selected and surface-decontaminated using 70% ethanol. Only visibly healthy and free of melanisation, larvae exhibiting consistent movement were included. Larvae that appeared sluggish, immobile, or discoloured were excluded from the study. All assays were independently repeated on three (for the burn only and burn infection control) or two (for the treatments and Tyr-AuNP control) separate occasions using 10 larvae per group per experiment (*n* = 160). Sample size was selected *a priori* based on published *G. mellonella* burn wound models.^25,26^ Larvae were randomly allocated into separate Petri dishes (10 larvae per plate) lined with Whatman filter paper (Fisher, UK) and housed in a static incubator at 37 °C until used. A burn wound was induced by heating a nail head in a blue Bunsen burner flame until red hot, cooled for 11 s and then superficially applied to achieve a wound area of approximately 2 mm^2^. Immediately post burn, the wound was inoculated with three to four colonies of *P. aeruginosa* PAO1 applied directly to the burn site. In a volume of 5 µL, the free AMPs and AMP-AuNPs were applied topically 1 h after infection at a final dosage of 10 mg kg^−1^ in 0.01 M PBS. The burn only control consisted of larvae with burn wounds that were not infected and received 5 µL of sterile 0.01 M PBS. The burn infection control group consisted of larvae with burn wounds infected with *P. aeruginosa* PAO1 and treated with 5 µL of sterile 0.01 M PBS instead of peptide. A Tyr-AuNP treatment control was also included where the Tyr-AuNP pellet was resuspended in the same volume as the AMP-AuNPs and 5 µL applied 1 h post infection. Survival was monitored over 144 h (6 days) and mortality recorded as complete melanisation of the larval body and complete loss of motility. *G. mellonella* survival was analysed by the log rank (Mantel–Cox) method and plotted as Kaplan–Meier survival curves.

### Data analysis

All *in vitro* assays were performed in triplicate, with at least two technical replicates per experiment (*n* = 6) and data were analysed using GraphPad Prism® v7.0. Statistical significance for both the TEM-based size analysis and the *in vitro* assays was assessed using ANOVA with Tukey's multiple comparisons test. Prior to ANOVA, data were evaluated for normality using the Shapiro-Wilk test. A *p*-value <0.05 was considered statistically significant in all analyses.

## Results and discussion

### Successful synthesis of AMP loaded AuNPs with high percentage loading efficiency

The rationale for loading the AMPs onto AuNPs to form AMP-AuNPs was to improve the proteolytic stability and reduce the potential cytotoxicity of the peptides. The parent peptide, AamAP1-Lys-NH_2_, was first modified to its all-d-enantiomer to enhance stability, then both the l- and d-forms were individually loaded onto AuNPs to evaluate the distinct and combined effects of enantiomerisation and gold-based delivery.

To enable efficient AMP loading, Tyr-capped AuNPs were first synthesised. Successful capping was indicated by a distinct colour change from yellow (tetrachloroauric acid) to ruby red (Tyr-AuNPs), as shown in Fig. 1A. Subsequent loading of AMPs onto the Tyr-AuNPs at a pH of 10.0 resulted in a further colour shift from ruby red to blue/purple, suggesting successful loading (Fig. 1A). This was confirmed quantitatively by changes in the SPR absorption bands, an optical property characteristic of metal nanoparticles (Fig. 1 and Fig. S1). SPR arises from the collective oscillation of conduction electrons on the nanoparticle surface when exposed to light. Shifts in SPR band intensity and wavelength are indicative of changes in particle size, shape, surface chemistry, the surrounding medium, or nanoparticle aggregation following loading.^27^ Therefore, alterations in the SPR profile reflect changes in the photophysical properties of the Tyr-AuNPs due to effective AMP loading.

The SPR bands of both Tyr-AuNPs and AMP-AuNPs were recorded in the 400–800 nm spectral range (Fig. 1B). Tyr-AuNPs exhibited a distinct SPR peak at 534 nm, consistent with previous reports for amino acid-capped AuNPs.^28,29^ Upon AMP loading, a broadening and red-shift of the SPR band to 560 nm was observed for both AamAP1-Lys-NH_2_-AuNP and d-AamAP1-Lys-NH_2_-AuNP, confirming surface modification associated with successful peptide loading.^29^

The percentage loading efficiency of AMPs onto the Tyr-AuNPs was determined using an indirect method involving UV-vis spectroscopy. After centrifugation to collect the AMP-AuNPs, the amount of unincorporated AMP remaining in the supernatant was measured. This approach allowed for accurate quantification of the loaded AMPs by calculating the difference between the initial AMP concentration and the residual amount in the supernatant. Subtraction of the absorbance at ∼215 nm of the prepared AamAP1-Lys-NH_2_-AuNP and d-AamAP1-Lys-NH_2_-AuNP supernatants from the absorbance peak of 400 µg mL^−1^ free/unreacted AMP, allowed the determination of the percentage loaded AMP. Findings show that ∼100% of the initial free AMP added to the Tyr-AuNP reaction mixtures successfully loaded to form the AMP-AuNPs (Fig. 1C and D, Table S1). Adjustment of the pH to 10.0 following the addition of Tyr-AuNPs to the dry AMPs was identified as a critical step for enhancing loading efficiency. When the pH is not adjusted to 10, <22% of AMP was successfully loaded (Fig. S2).

To further investigate the influence of pH on AMP loading and nanoparticle aggregation, peptide loading was evaluated across pH ranges of 4–5, 6–7, 8–9, and 10–11 (Fig. S3). SPR analysis demonstrated progressive increases in SPR peak intensity and peak broadening with increasing pH, indicating enhanced peptide adsorption and changes in nanoparticle interactions (Fig. S3). At pH 4–5, the SPR spectrum became substantially flattened with the absence of a distinct SPR peak, suggesting destabilisation of the Tyr-capped AuNP system under acidic conditions. This may be due to protonation and partial loss of the Tyr capping layer from the AuNP surface, resulting in reduced colloidal stability, poor peptide loading, and minimal observable aggregation. Correspondingly, stereomicroscopy revealed little to no visible aggregate formation under these conditions (Fig. S3). At pH 6–7, moderate SPR shifts were observed together with the appearance of small aggregates. More pronounced aggregation occurred at pH 8–9, where larger aggregate structures were visible both macroscopically and microscopically. Interestingly, at pH 10–11, although peptide loading and SPR intensity were highest, the aggregates appeared smaller and more uniform in morphology. These findings suggest that pH plays a critical role in regulating both peptide adsorption and nanoparticle aggregation.

The mechanism by which AMP loading may induce aggregation is not yet fully understood, but several plausible explanations exist, supported by findings from other studies^43^ and our own observations. Amphipathic AMPs such as AamAP1-Lys-NH_2_ and d-AamAP1-Lys-NH_2_ may aggregate on the surface of Tyr-AuNPs through hydrophobic interactions between their non-polar residues. This tendency is consistent with a previous study showing that free AamAP1-Lys-NH_2_ can self-associate *via* its hydrophobic domains, leading to aggregation.^23^ Alternatively, aggregation may also occur *via* electrostatic interactions. In a study by Ma *et al.*,^44^ a polymer was added to citrate-capped AuNPs (which carry a negative surface charge). Upon exposure to CO_2_, the polymer became cationic, leading to aggregation through electrostatic attraction between the positively charged polymer and the negatively charged AuNP surface.^45,57^ A similar mechanism may occur in the Tyr-AuNP system, where increasing pH promotes progressive deprotonation of the phenolic hydroxyl group of tyrosine residues, resulting in a more negatively charged nanoparticle surface. This enhances electrostatic interactions with the positively charged lysine residues of AamAP1-Lys-NH_2_ and d-AamAP1-Lys-NH_2_, leading to increased peptide adsorption.

At intermediate pH values, partial surface deprotonation may favour interparticle bridging and irregular aggregate formation, whereas at pH 10–11, greater surface charge and peptide coverage may promote the formation of smaller, more uniform aggregates. In addition, the aromatic ring of tyrosine may contribute to hydrophobic and π-related interactions with aromatic or hydrophobic residues within the peptides, further stabilising peptide association with the nanoparticle surface. These combined interactions may therefore explain the enhanced peptide loading observed under alkaline conditions.

Aggregation may additionally arise from reduced electrostatic repulsion between nanoparticles. It is likely that while the positively charged residues of the AMPs interact with the negatively charged Tyr-AuNP surface, the hydrophobic and neutral residues orient outward toward the surrounding aqueous environment. This outward orientation may effectively shield the nanoparticle's negative surface charge, reducing electrostatic repulsion between particles and thereby promoting aggregation.^46^

The high loading efficiency of AMPs onto AuNPs at pH 10.0 prompted further investigation into the surface chemistry of the Tyr-AuNPs and AMP-AuNPs.

### FTIR and confocal microscopy confirm AMP adsorption onto AuNP surfaces

**Fig. 2:**
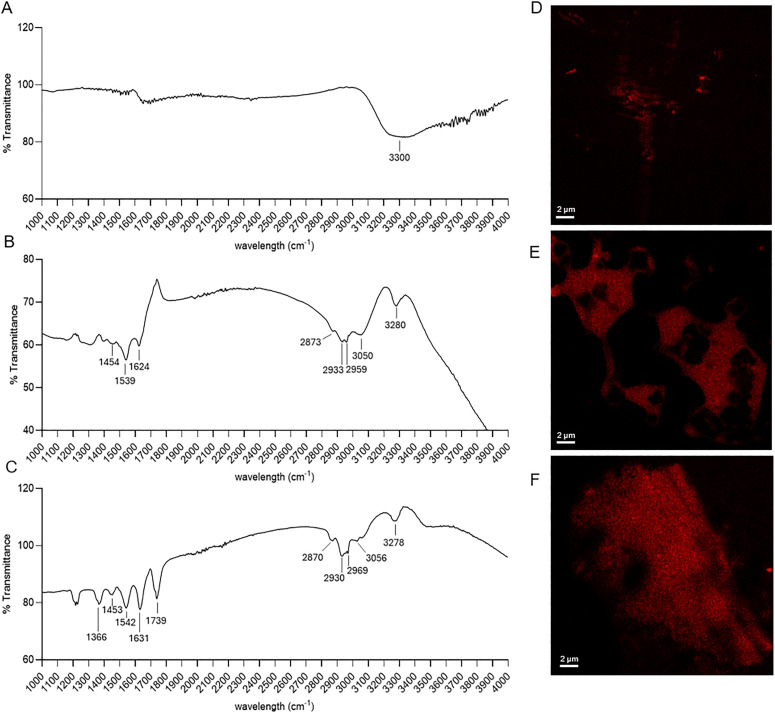
Surface of AuNPs changes after loading of AMPs. Characterisation of the surface of the Tyr-AuNP and AMP-AuNPs using FTIR spectroscopy and confocal microscopy. One representative of two independent repeats of the ATR-FTIR spectra of (A) Tyr-AuNPs, (B) AamAP1-Lys-NH_2_-AuNPs and (C) d-AamAP1-Lys-NH_2_-AuNPs with 4 cm^−1^ resolution recorded between 1000–4000 cm^−1^. One representative of duplicate confocal fluorescence images of (D) Tyr-AuNPs, (E) AamAP1-Lys-NH_2_-AuNPs and (F) d-AamAP1-Lys-NH_2_-AuNPs excited at *λ* = 514 nm, shown with a scale = 2 µm.

The functional groups present on the surface of the Tyr-AuNP and AMP-AuNPs (Fig. 2A–C and S5), as well as those present in free AamAP1-Lys-NH_2_ and d-AamAP1-Lys-NH_2_ (Fig. S4A and B), were investigated with ATR-FTIR spectroscopy. The spectra of all AuNPs and free peptides were recorded in the range of 1000–4000 cm^−1^. The FTIR spectrum of Tyr-AuNP showed a single, broad OH stretching band at 3300 cm^−1^, indicating the presence of the hydroxyl group of Tyr (Fig. 2A). No other characteristic vibrational bands were detected in the Tyr-AuNP spectrum, confirming the simplicity of its surface chemistry and the absence of additional organic functional groups prior to AMP incorporation.

The FTIR spectra of AamAP1-Lys-NH_2_-AuNP and d-AamAP1-Lys-NH_2_-AuNP showed multiple vibrational bands corresponding to various amino acid side chains which confirm the presence of AMPs on the AuNP surfaces. The bands observed at 3280 cm^−1^ for AamAP1-Lys-NH_2_-AuNP and 3278 cm^−1^ for d-AamAP1-Lys-NH_2_-AuNP, are assigned to the OH stretching vibrations of the serine (Ser) hydroxyl group (Fig. 2B and C). The vibrational frequency bands at 3050 cm^−1^ (AamAP1-Lys-NH_2_-AuNP) and 3056 cm^−1^ (d-AamAP1-Lys-NH_2_-AuNP) correspond to the aromatic C–H stretching vibrations of phenylalanine (Phe) residues. Additional bands observed at 2959/2969 cm^−1^, 2933/2930 cm^−1^, and 2873/2870 cm^−1^ are attributed to C–H stretching vibrations of methyl (–CH_3_) groups in leucine (Leu), isoleucine (Ile), and alanine (Ala). The presence of lysine (Lys) side chains is indicated by the –NH_3_^+^ stretching bands at 1624/1631 cm^−1^. Furthermore, the bands at 1539/1542 cm^−1^ and 1454/1453 cm^−1^ are consistent with aromatic C=C–C stretching of Phe residues. The 1454/1453 cm^−1^ bands may also partially overlap with the C–N stretching vibrations of proline (Pro).^30,31^

Collectively, these spectral features confirm the presence of the amino acid side chains within the AMP structures. Notably, the characteristic vibrational bands observed in the FTIR spectra of the free peptides (Fig. S4A and B) are retained in the spectra of the AMP-AuNPs, providing strong evidence for the successful surface loading of the AMPs onto the Tyr-AuNPs.

Confocal fluorescence microscopy was used in support of FTIR analysis to evaluate the AuNP surface chemistry (Fig. 2D–F and Fig. S5). The intrinsic fluorescence of aromatic amino acids, upon excitation with a green laser (514 nm), was utilised to visualise the presence of Tyr on the AuNP surface, as well as to confirm AMP incorporation. Increased red fluorescence was visually observed in the AMP-AuNP samples compared to the Tyr-AuNPs, indicating a higher concentration of amino acids in the AMP-functionalised samples. This qualitative observation supports the successful and dense loading of AMPs onto the Tyr-AuNPs and suggests that Tyr is not only present but may also facilitate AMP adhesion, potentially acting as a linker.

Previous studies have shown that linker molecules are not always necessary, as AMPs themselves can function as both reducing and capping agents, thereby simplifying the synthesis of AMP-functionalised AuNPs. However, this approach depends heavily on the reducing and binding capabilities of specific amino acid residues, particularly tryptophan (Trp) and Lys, which are known to exhibit strong activity in this context.^32^ Peptides lacking these key residues often result in poor or failed nanoparticle formation. These capabilities can be further influenced by the overall sequence, conformation, and the positioning of other nearby residues.

In the early stages of this study, attempts were made to synthesise AamAP1-Lys-NH_2_-AuNPs using AamAP1-Lys-NH_2_ alone as both reducing and capping agent. Despite the presence of six Lys residues, these efforts were unsuccessful (data not shown), suggesting that AamAP1-Lys-NH_2_ alone lacks sufficient reducing or binding capacity under the conditions tested. However, when the modified two-step method was employed by first reducing and capping the AuNPs with Tyr, followed by AMP addition at specific pH levels (10.0), complete (∼100%) peptide loading was consistently achieved, demonstrating the effectiveness of Tyr as a stabilising and functional intermediary for AMP attachment.

The synthesis approach used in this study enables the formation of AMP-AuNPs *via* a Tyr-AuNP intermediate and relies solely on the cationic nature of AMPs, a common feature among most AMPs. As such, this method has the potential to be applied broadly across different AMPs and could serve as a universal synthesis approach to improve peptide stability, reduce cytotoxicity, and support the development of AMP-based antibacterial therapeutics.

### AMP loading onto AuNPs induces aggregation

Particle size significantly influences the biological activity of AuNPs, influencing both their antibacterial efficacy and cytotoxicity.^34,35^ Generally, smaller AuNPs (<20 nm) tend to exhibit enhanced antibacterial activity due to their increased surface area and reactivity, which can lead to greater cellular uptake and oxidative stress. This increased activity is often accompanied by higher cytotoxicity as well.^36^ However, the relationship between smaller particle size and increased antibacterial or cytotoxic effects is not consistently observed. Several studies report that smaller AuNPs do not necessarily result in improved antibacterial performance^37^ or elevated cytotoxicity.^38^ These inconsistencies suggest that, beyond particle size, other factors such as nanoparticle morphology and surface functionalisation might also play important roles in determining biological outcomes.

**Fig. 3:**
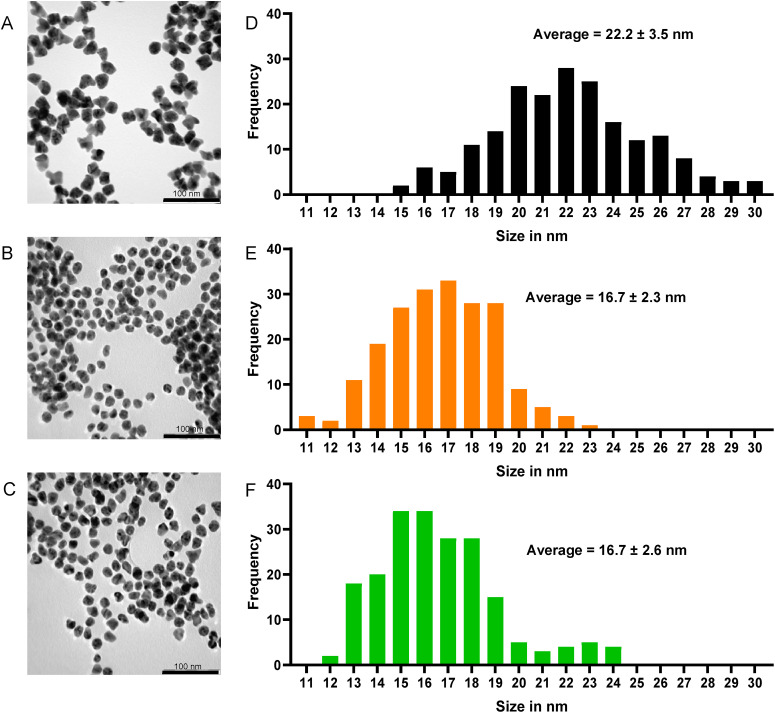
Morphology and size distribution of Tyr-AuNPs and AMP-AuNPs. TEM images of (A)–(C) and corresponding size distribution histograms (D)–(F) for Tyr-AuNP (A) and (D), AamAP1-Lys-NH_2_-AuNP (B) and (E) and d-AamAP1-Lys-NH_2_-AuNP (C) and (F) show the morphology of the AuNPs with scale bars of 100 nm and the average AMP-AuNP size ± standard deviation determined by measuring the diameter of one hundred Tyr-AuNPs and AMP-AuNPs in each sample, on two different occasions (*n* = 200) using ImageJ v1.6 software. Size distribution was obtained using histogram frequency distribution analysis of the measured particle diameters.

Here, the size and morphology of the Tyr-AuNPs and the AMP-AuNPs, formed at pH 10.0, were determined using TEM (Fig. 3A–F). Both the Tyr-AuNPs and AMP-AuNPs exhibited quasi-spherical shapes (Fig. 3A–C), however the Tyr-AuNPs showed more irregular morphology, with some nanostar-like structures, characterised by asymmetric, branched, or semi-spiked features^33^ (Fig. 3A). The average diameter of the Tyr-AuNPs was 22.2 ± 3.5 nm, while the AamAP1-Lys-NH_2_-AuNPs and d-AamAP1-LysNH_2_-AuNPs displayed smaller and similar average diameters of 16.7 ± 2.3 nm and 16.7 ± 2.6 nm, respectively (Fig. 3E and F and Fig. S6). The significantly larger size of the Tyr-AuNPs is likely related to the gradual and continued growth of the gold nuclei forming these nanostar-like morphologies, whereas the growth of the nuclei is halted in AamAP1-Lys-NH_2_-AuNPs and d-AamAP1-Lys-NH_2_-AuNPs due to AMP loading. This phenomenon is commonly reported across various synthesis methods for drug-loaded AuNPs.^21^

Interestingly, the SPR bands observed for the AMP-AuNPs (Fig. 1) did not correlate with their measured core sizes (Fig. 3). Typically, for well-dispersed AuNPs, an increase in core size results in a red shift, *i.e.* a shift to higher wavelengths.^39^ However, despite TEM images showing smaller core sizes (16 to 17 nm) for the AMP-AuNPs formed at pH 10.0, their SPR maxima were red-shifted beyond the 534 nm peak of the larger Tyr-AuNPs, reaching 560 nm. This discrepancy suggests that other factors may influence the SPR behaviour, with aggregation of the AMP-AuNPs leading to plasmon coupling and overlapping of electronic clouds, being a plausible explanation. Aggregated AMP-AuNPs are potentially behaving like larger AuNPs and are associated with a dark purple/blue colour and a shift in the SPR maximum to a higher wavelength.^39^

Generally, aggregation is an outcome to be avoided during the synthesis of AuNPs, as it can negatively affect nanoparticle stability and function.^40,41^ However, here, the AMP-AuNPs formed at pH 10.0 produced relatively small and uniform aggregates while simultaneously achieving exceptionally high peptide loading efficiencies. Previous studies often report loading efficiencies below 50%,^42^ and in such cases, aggregation can further reduce the amount of peptide available for release.^40–42^ In contrast, the high loading achieved here suggests that even with some degree of aggregation, a sufficient amount of AMP remains accessible, potentially preserving therapeutic efficacy. Importantly, the potential limitations associated with aggregation may be further mitigated by the ability of these aggregates to dissociate under physiologically relevant conditions.

To investigate this possibility, AMP-AuNP aggregates formed at pH 10.0 were resuspended in sterile ddH_2_O at pH 7.0 and PBS at pH 7.4 to evaluate the influence of neutral pH and physiological salt conditions on aggregate stability (Fig. S7). Stereomicroscopy revealed that, in water, large aggregates were present immediately following resuspension (0 min). Partial reduction in aggregate size was observed after 10 min, indicating some degree of dissociation, with little additional change observed up to 60 min. In contrast, AMP-AuNPs resuspended in PBS displayed substantially smaller aggregates at the initial 0 min time point compared with those in water (Fig. S7). Further dissociation occurred over the subsequent 10–30 min period, and by 60 min, aggregates were barely visible even at higher magnifications. These findings suggest that although aggregation occurs during the high-pH peptide loading process, the aggregates are not irreversibly stable and gradually dissociate under neutral, salt-containing physiological conditions. This behaviour may therefore overcome many of the concerns typically associated with nanoparticle aggregation by promoting nanoparticle dispersion and gradual peptide release following administration in physiological environments such as wounds.

### Release of AMPs from the nano-gold delivery systems are tuneable by varying salt concentration

For AMP-AuNPs to be therapeutically effective, it is crucial that the loaded peptides are released under physiological conditions. However, given the initial aggregation observed in the AMP-AuNP formulations in this study, together with their gradual dissociation under physiologically relevant conditions, these systems may be particularly well suited for topical applications such as the treatment of infected wounds or soft tissue infections. In such environments, localised delivery and sustained peptide release may be advantageous, while extensive systemic circulation is not required.

Moreover, combining drug delivery systems with solutions that promote wound healing could further enhance their therapeutic potential. For instance, saline solutions commonly used in wound care have been shown to aid healing through osmotic effects.^47^ Therefore, pairing AMP-AuNPs with a saline-based solution like PBS for peptide release into infected wounds would be advantageous.

**Fig. 4:**
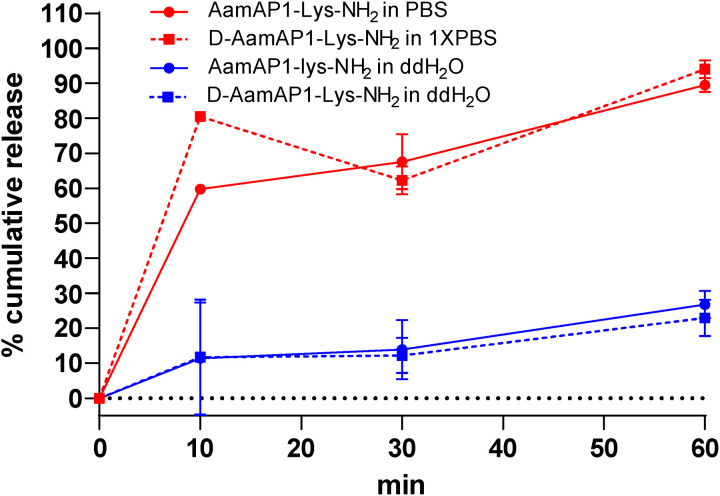
AMPs are gradually released from the AMP-AuNPs. The release of peptides from the AuNPs in 1× PBS (pH 7.4) and in ddH_2_O (pH 7.0) over 60 min are presented as the mean percentage cumulative release with standard error of the mean (SEM) shown from two independent repeats (*n* = 2). AMP release was quantified by UV-vis spectroscopy by monitoring the absorbance of AMP-AuNP supernatants at 215 nm. Percentage cumulative release was calculated by normalising the supernatant absorbance to that of a 200 µg mL^−1^ free AMP solution, which was defined as 100% release, with PBS or ddH_2_O used as blanks.

Accordingly, the release profiles of AMPs from AMP-AuNPs were evaluated at a more neutral pH, both in the absence and presence of salt ions, over a 60 min period (Fig. 4). When resuspended in ddH_2_O in the absence of salt (pH 7.0), approximately 10% of the peptides were released from both AamAP1-Lys-NH_2_-AuNPs and d-AamAP1-Lys-NH_2_-AuNPs within the first 30 min. This gradually increased to around 20% by 60 min, indicating a slow and sustained release under these conditions (Fig. 4).

This corresponds with the stereomicroscopy-based dissociation studies, where only a slight reduction in aggregate size was observed over time in water, suggesting that minimal aggregate dissociation may accompany the slow release of peptides under low-ionic-strength conditions (Fig. S7).

The slow but gradual release may be attributed to the weakening of electrostatic interactions between the AMPs and the Tyr-AuNPs at neutral pH, possibly due to partial protonation of the Tyr hydroxyl and amino groups. Another plausible explanation is the dissociation of the Tyr layer from the AuNP surface, resulting in the release of AMPs either directly or as part of a Tyr-AMP complex. Regardless of the exact mechanism, the data suggest that under low-ionic-strength, physiological pH conditions, the AMP-AuNPs provide a slow and sustained release of the peptides.

Next, the release profiles were evaluated in the presence of salt ions to assess the feasibility of combining the AMP-AuNP delivery system with saline solutions for wound treatment. Notably, when the AMP-AuNPs were suspended in PBS containing 0.01 M salt ions, peptide release was substantially faster than in ddH_2_O. Within the first 10 min, approximately 60% of both AamAP1-Lys-NH_2_ and d-AamAP1-Lys-NH_2_ were released from the respective AMP-AuNPs (Fig. 4). At 30 min, this amount remained similar with perhaps an additional 5% of AMP released. However, after 60 min another ∼30–35% of AMP is released with a 90–100% cumulative release. This indicates an initial burst release of AMPs in PBS in the first 10 min compared to water, followed by a slower, sustained release phase over the subsequent 50 min. This release behaviour correlates with the stereomicroscopy-based dissociation studies performed in PBS, where aggregate size rapidly decreased following resuspension and became barely visible over time, suggesting that peptide release may occur concomitantly with aggregate dissociation under salt-containing physiological conditions (Fig. S7).

The enhanced release observed in PBS is likely due to a combination of weakened electrostatic interactions at neutral pH, as described above, together with additional ionic disruption caused by the salt ions. The salt ions may interfere with the interactions between the cationic AMPs and the negatively charged Tyr-AuNP surface by shielding or competing with positively charged amino acid residues, thereby facilitating peptide detachment and nanoparticle dissociation.

The gradual release observed in water, alongside the initial burst followed by sustained release in the presence of salt ions, indicates that the AMP-AuNP release profiles are highly tuneable. This tunability may be advantageous for optimising delivery strategies in future therapeutic applications. Furthermore, sustained release of AMPs may protect them against the effect of proteases for longer and ensure an increased period of dosing compared with the administration of a single dose of free AMPs.

### Antipseudomonal potency is retained upon release of AMPs from the AuNP delivery system

The key features of the developed AMP-AuNP delivery system include pH optimisation and electrostatic binding, enabling 100% loading efficacy *via* a tyrosine linker and allowing a tuneable AMP release mechanism. The key question is whether these features add value to the development of the AMP-AuNP delivery system for the treatment of wound infections. Therefore, the AMP-AuNPs were subsequently evaluated for *in vitro* antipseudomonal activity against *P. aeruginosa* PAO1 and compared with free AMPs to confirm that released peptide from AuNPs remain active (Table 1).

**Table 1:** The antibacterial activity of the AMPs and AMP-AuNPs (in µg mL^−1^) against *P. aeruginosa* PAO1 after 1 h pre-treatment with trypsin and FCS

Treatment		Peptides		AMP-AuNP	
		AamAP1-Lys-NH_2_	d-AamAP1-Lys-NH_2_	AamAP1-Lys-NH_2_-AuNP	d-AamAP1-Lys-NH_2_-AuNP^a^
None	MIC	4	4	9–17	9–17
	MBC	4	17	17	17–35
2.5 mM	MIC	>138	—a	**17–35**	—b
Trypsin	MBC	>138	—a	**17–35**	—b
5 mM	MIC	>138	**4**	69–138	—b
Trypsin	MBC	>138	**17**	138	—b
10% FCS	MIC	**4–9**	**4–9**	**9–17**	—b
	MBC	9–17	**9–17**	**9–17**	—b

a Stability of d-AamAP1-Lys-NH_2_ not determined at lower trypsin concentration as the peptide remains stable when pre-treated with 5 mM trypsin.

b Stability of d-AamAP1-Lys-NH_2_-AuNP not evaluated as d-AamAP1-Lys-NH_2_ is stable in trypsin and FCS.

The free AMPs showed potent antimicrobial activity with low MICs of 4 µg mL^−1^ and equally low MBCs of 4 µg mL^−1^ and 17 µg mL^−1^ for AamAP1-Lys-NH_2_ and d-AamAP1-Lys-NH_2_, respectively. The slight increase in MBC observed for d-AamAP1-Lys-NH_2_ is most likely attributed to commonly reported changes in peptide conformation or membrane interaction dynamics upon d-amino acid substitution.^10,11,48^ Indeed, it was found that AamAP1-Lys-NH_2_-AuNP and d-AamAP1-Lys-NH_2_-AuNP retained activity with low MICs in the range of 9–17 µg mL^−1^ and MBCs of 17–35 µg mL^−1^, while the unloaded Tyr-AuNP control showed no inhibitory activity (data not shown). These results indicate that both peptides remain functionally active when gradually released from the AuNP surface in salt-containing MHB media, with the slightly reduced apparent activity of the peptide-loaded AuNPs likely attributable to slower release from the nanoparticle surface compared to the immediate availability of free peptides.

Numerous studies have shown that the size of AuNPs can significantly influence their antibacterial performance, with smaller particles (<25 nm) generally associated with lower MICs and MBCs compared to larger AuNPs (60–90 nm).^47^ The AamAP1-Lys-NH_2_-AuNP and d-AamAP1-Lys-NH_2_-AuNPs produced in this study fall within this optimal size range (16.7 nm), where effective antibacterial activity and low cytotoxicity are typically observed.

It is also important to consider the role of aggregation. While aggregation of AuNPs is often regarded as undesirable due to potential instability, reduced surface area and altered biodistribution,^40,41^ the findings of the present study suggest that the observed aggregation may not necessarily be detrimental. Although the AMP-AuNPs formed at pH 10.0 initially displayed small, relatively uniform aggregates with high peptide loading efficiencies, the dissociation studies demonstrated that these aggregates are not permanently stable and gradually dissociate under neutral, salt-containing physiological conditions. This dissociation behaviour correlates with the peptide release studies, with burst initial peptide release followed by sustained release observed in the presence of salts.

These findings suggest that aggregation may be a transient structural state formed during high-pH peptide loading, rather than irreversible nanoparticle instability. Such transient aggregation may provide functional advantages by initially protecting surface-bound peptides and subsequently enabling gradual nanoparticle dissociation and peptide release over time under physiological conditions. Similar concepts of tuneable or responsive nanoparticle aggregation have been proposed as strategies to improve therapeutic performance.^49–52^ Therefore, in the context of AMP-AuNP delivery systems, controlled aggregation followed by physiologically triggered dissociation may represent a beneficial feature.

### Loading of AamAP1-Lys-NH_2_ onto AuNPs improves proteolytic stability

The stability of the free l-amino acid peptide was first compared to its all-d-enantiomer, and subsequently to the AMP-AuNPs, to determine which strategy conferred the greatest improvement in proteolytic stability. Trypsin and serum stability of the AMPs were examined by pre-treatment with 2.5 or 5 mM trypsin and 10% FCS, followed by MIC and MBC determination (Table 1). After treatment with either 2.5 or 5 mM trypsin, free AamAP1-Lys-NH_2_ lost all activity against *P. aeruginosa* PAO1, indicating instability in the presence of trypsin. In contrast, the MIC and MBC of the all-d-enantiomer remained unchanged at 4 and 17 µg mL^−1^, respectively, confirming its stability under enzymatic conditions. Both AamAP1-Lys-NH_2_ and d-AamAP1-Lys-NH_2_ retained antibacterial activity after pre-treatment with 10% FCS, with only slight increases in MICs and MBCs, which remained low at 4–9 µg mL^−1^ and 9–17 µg mL^−1^, respectively.

While free AamAP1-Lys-NH_2_ lost all activity in the presence of trypsin, the AamAP1-Lys-NH_2_-AuNP offers moderate protection against proteolytic degradation, maintaining antimicrobial activity with slightly increased MIC and MBC values of 17–35 µg mL^−1^ after exposure to 2.5 mM trypsin. Although activity was further reduced following treatment with 5 mM trypsin, AamAP1-Lys-NH_2_-AuNPs remained active, with an MIC range of 69–138 µg mL^−1^ and an MBC of 138 µg mL^−1^. This retained activity may be explained by a dual protective mechanism: (i) a fraction of AMP remains surface-associated and is sterically shielded by adsorption onto the AuNP surface, limiting direct access of trypsin to peptide substrates, and (ii) a gradual release of AMP from the nanoparticle surface replenishes active peptide in solution over time, sustaining antimicrobial activity even under proteolytic conditions. These results demonstrate that although the AMP-AuNP conferred improved proteolytic stability compared to the free peptide, the d-enantiomer is more effective at resisting enzymatic degradation.

Combining the two formulation strategies by loading d-AamAP1-Lys-NH_2_ onto AuNPs is expected to provide optimal proteolytic and serum stability, as free d-AamAP1-Lys-NH_2_ retained full antimicrobial activity after enzymatic challenge. However, the primary rationale for loading d-AamAP1-Lys-NH_2_ onto AuNPs is to mitigate the possible cytotoxicity associated with the d-enantiomer, a well-established advantage of nanogold-based delivery systems. Consequently, the next step was to evaluate the impact of AuNP loading on the cytotoxicity of the AMPs.

### Loading of d-AamAP1-lys-NH_2_ onto AuNPs reduces cytotoxicity

**Fig. 5:**
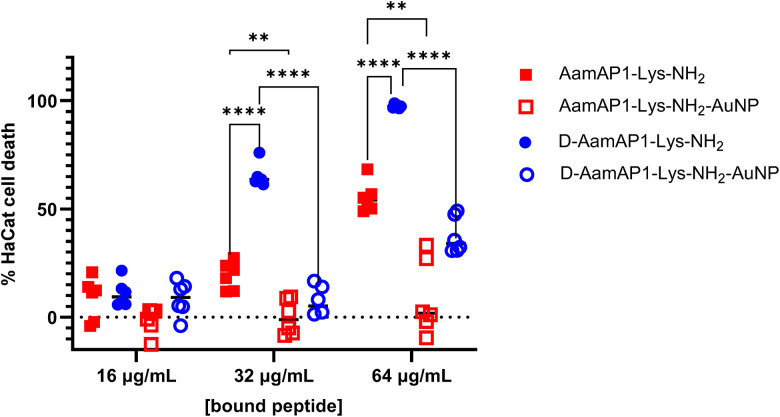
AMP-AuNPs reduce HaCat cytotoxicity compared with free AMPs. Cytotoxicity of free AMPs and AMP-AuNPs was assessed after 24 h treatment of HaCat cells with varying concentrations of bound peptide. Each condition was tested in three independent experiments, each performed in duplicate. Statistical significance was determined using two-way ANOVA with Tukey's multiple comparisons test. Significance is indicated as follows: **** = *p* < 0.0001, *** = *p* < 0.0005, ** = *p* < 0.005, * = *p* < 0.05.

The cytotoxicity of the free AMPs and AMP-AuNPs as well as their potential for topical application was evaluated using HaCat cells. At 32 and 64 µg mL^−1^, the d-AamAP1-Lys-NH_2_ enantiomer is significantly more cytotoxic than AamAP1-Lys-NH_2_ causing 73.1 ± 11.5% and 97.0 ± 1.0% cell death *versus* 19.1 ± 6.3% and 55.5 ± 7.0% cell death caused by AamAP1-Lys-NH_2_ (Fig. 5).

Loading d-AamAP1-Lys-NH_2_ onto AuNPs resulted in a substantial reduction in cytotoxicity toward HaCat cells, with cell death decreasing to 8.5 ± 8.0%, 4.5 ± 11.4%, and 37.7 ± 8.4% at concentrations of 16, 32, and 64 µg mL^−1^, respectively. Similarly, loading of AamAP1-Lys-NH_2_ onto AuNPs significantly reduced cytotoxicity at 64 µg mL^−1^, lowering cell death from 55.5 ± 7.0% for the free peptide to just 8.7 ± 17.2% following nanoparticle loading.

The decrease in cytotoxicity observed is likely related to the gradual release of the AMPs from the AuNPs. Free peptides are known to act rapidly, typically within minutes,^6^ and are therefore fully bioavailable immediately upon administration, which may contribute to their higher observed toxicity. In contrast, peptides loaded onto AuNPs are released in a controlled manner over time. Release studies conducted in PBS demonstrated an initial release of approximately 60–80% AMP over the first 10 minutes, followed by a plateau up to 30 minutes, and a more gradual release thereafter.

These findings suggest that ionic strength plays an important role in facilitating peptide release from the gold surface. Given that DMEM similarly contains a high concentration of salts, comparable release behaviour is likely under biological conditions. As a result, peptide–AuNP loading reduces the immediate availability of the peptide, thereby lowering its acute cytotoxic effects. This is consistent with the 97% toxicity observed for the free d-AamAP1-Lys-NH_2_ peptide at 64 µg mL^−1^ which decreased to 40% when gradually released from AuNPs.

Overall, both AMP-AuNPs have advantages regarding increased proteolytic stability and reduced cytotoxicity, even though *in vitro* potency is not enhanced. Subsequently, it was determined whether the added benefits of the AMP-AuNP delivery systems translate to *in vivo* topical application, specifically for the treatment of infected burn wounds.

### Protection of larvae against *P. aeruginosa* PAO1 wound infection in an *in vivo G. mellonella* burn wound model

The AMP-AuNPs were evaluated and compared with their free AMP counterparts for their ability to enhance therapeutic outcomes in *P. aeruginosa*-infected *G. mellonella* larvae using an *in vivo* burn wound model. Although the *G. mellonella* infection model is widely accepted for preliminary *in vivo* antimicrobial assessment, it does not fully replicate the complexity of mammalian wound environments. In particular, differences in immune system organisation, skin structure, and wound healing processes limit direct extrapolation to mammalian systems. Nevertheless, *G. mellonella* provides an ethically accessible intermediate model that allows rapid *in vivo* screening of antimicrobial efficacy prior to evaluation in more complex mammalian infection and wound healing models.

**Fig. 6:**
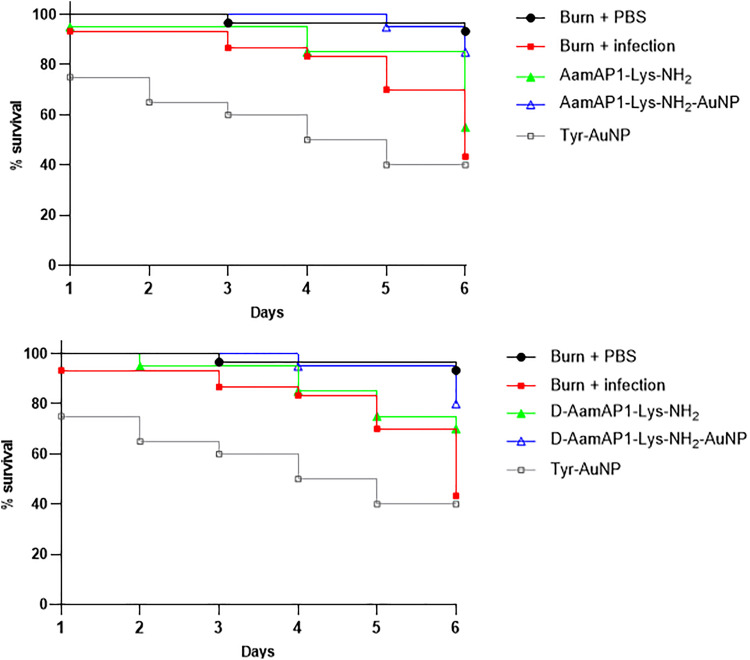
AMP-AuNPs protect *G. mellonella* larvae with *P. aeruginosa* PAO1 burn wound infection. Survival curves were generated for 20 larvae per treatment group receiving a single 10 mg kg^−1^ dose of AamAP1-Lys-NH_2_, AamAP1-Lys-NH_2_-AuNP (A) or d-AamAP1-Lys-NH_2_, d-AamAP1-Lys-NH_2_-AuNP (B) and compared to 30 larvae per group subjected to burn-only or burn plus infection controls over a 6-day period. Tyr-AuNP treatment of 20 larvae was added as an additional control. Percentage survival and the significance of protection against infection due to therapy, according to Log-rank (Mantel–Cox) and Gehan–Breslow–Wilcoxon test, are summarised in Table 2.

Larvae that received burn wounds only, with no infection and only PBS treatment survived with no significant mortality. Subsequent infection with *P. aeruginosa* PAO1 led to significant mortality with only 43.3% survival (Fig. 6 and Table 2).

**Table 2:** The percentage survival of burn wound infected (*P. aeruginosa* PAO1) *G. mellonella* larvae at day 6 after treatment with 10 mg kg^−1^ dosages of AMP and AMP-AuNP

Condition	Survival % at 144 h	*P*-Value (*P* < 0.05)	
		Log-rank (Mantel–Cox) test	Gehan–Breslow–Wilcoxon test
Burn only	93	<0.0001	<0.0001
Burn + infection	43	—	—
Tyr-AuNP	40	0.2479	0.0990
AamAP1-Lys-NH_2_	55	0.3346	0.3269
d-AamAP1-Lys-NH_2_	70	0.1147	0.1489
AamAP1-Lys-NH_2_-AuNP	85	*0.0033* a	*0.0033* a
		*0.0406* b	*0.0389* b
d-AamAP1-Lys-NH_2_-AuNP	80	*0.0091* a	*0.0088* a
		0.3849^b^	0.3445^b^

a The *p*-values indicate significant differences relative to the burn + infection control.

b The *p*-values of the AMP-AuNP *versus* the free AMP counterpart values in italics indicate significant differences relative to AamAP1-Lys-NH_2_.

Although free AamAP1-Lys-NH_2_ showed potent activity *in vitro*, it did not significantly improve infected larvae survival *in vivo* (55.0%), likely due to its cytotoxicity and susceptibility to proteolytic degradation (Table 2). In contrast, the all-d-enantiomer resulted in a greater, albeit statistically non-significant, improvement in survival (70.0%), with a trend suggesting enhanced efficacy that may become significant with larger treatment groups (*e.g. n* ≥ 30), potentially due to its increased resistance to proteases commonly present in wound environments (Table 2).

Upon loading both these AMPs onto AuNPs, a significant increase in the survival of larvae with infected burn wounds was recorded with 85.0% (*p* = 0.0033) and 80.0% (*p* = 0.0091) for AamAP1-Lys-NH_2_-AuNP and d-AamAP1-Lys-NH_2_-AuNP respectively, compared with the infected burn control (Fig. 6 and Table 2).

The AamAP1-Lys-NH_2_-AuNP significantly improved the survival of larvae compared with its free AMP counterpart, with a difference of 30.0% (*p* = 0.0406) between the two treatments groups. This improvement is likely due to the gradual release of AMPs and the improved stability afforded by the nanogold delivery system. In contrast, the slight improvement observed for the larvae treated with d-AamAP1-Lys-NH_2_-AuNP compared with the free d-AamAP1-Lys-NH_2_ (10.0% difference), although not statistically significant, is likely attributable to reduced toxicity conferred by the AuNP delivery system, as d-AamAP1-Lys-NH_2_ is already stable against proteolytic degradation (Table 2). The true therapeutic potential of d-AamAP1-Lys-NH_2_ coated gold is likely to become clearer through larger-scale studies and further clinical testing.

Interestingly, Tyr-AuNPs alone offered no protection against *P. aeruginosa* PAO1 burn wound infections in *G. mellonella* larvae, as indicated by the lack of a significant difference in survival compared to the burn and infection control group (*p* = 0.2479 and *p* = 0.0990, respectively), highlighting that the protective effect arises from the loaded AMPs rather than the Tyr-AuNPs.

Overall, these findings demonstrate the potential of this AuNP-based delivery system to overcome common limitations associated with AMPs, specifically instability and cytotoxicity, and align with previous reports on the efficacy of AMP-functionalised AuNPs.^21,53^

## Conclusion

The treatment of infected wounds, particularly those caused by MDR pathogens, remains a significant clinical challenge. While AMPs offer a promising alternative to conventional antibiotics, their therapeutic application is often limited by proteolytic degradation and host toxicity. In this study, we addressed these limitations through a novel nanogold-based delivery system, specifically by loading the synthetic AMP AamAP1-Lys-NH_2_ and its all-d-enantiomer onto AuNPs using a unique pH-dependent strategy.

This work demonstrates an optimised AMP-to-AuNP ratio at pH 10.0, enabling 100% loading efficiency and complete peptide release within 60 min at physiological pH (7.4). Release was shown to be accelerated in PBS compared to water, simulating wound environments, suggesting relevance for real-world application. Notably, both l- and d-AamAP1-Lys-NH_2_-AuNP conjugates exhibited reduced cytotoxicity to HaCat cells and significantly improved *in vivo* efficacy, as evidenced by enhanced survival of *P. aeruginosa* PAO1-infected *G. mellonella* larvae. The d-form peptide, while more resistant to proteases, showed higher toxicity in free form, yet this was mitigated by AuNP loading, likely due to the gradual peptide release from the nanoparticles, resulting in lower effective peptide concentrations at any given time.

The novelty of this study lies not only in the promising therapeutic potential of AamAP1-Lys-NH_2_-AuNP for future clinical development especially for the treatment of infected wounds, but also in the optimised formulation strategy that enables reliable and reproducible peptide loading and release without the need for linkers or cysteine residues, addressing a challenge in nanoparticle-based AMP delivery.

Future work will focus on broadening this platform by exploring a wider range of AMPs with diverse structural and functional properties, refining the AuNP loading parameters (including peptide-to-nanoparticle ratios and surface chemistries), and systematically characterising release kinetics under physiologically relevant conditions, including various saline concentrations and enzymatic environments. Further *in vivo* studies in mammalian wound infection models will be essential to validate safety, efficacy, and biodistribution, and to perform dose–response assessments to determine whether higher doses of the AMP-AuNP remain well tolerated and could potentially achieve complete survival. Overall, this study lays a strong foundation for the rational design and translation of the AMP-AuNP delivery system into clinically viable treatments for MDR wound infections.

## Author contributions

Rosalind J. Van Wyk: conceptualisation, methodology, formal analysis, investigation, writing – original draft preparation, visualisation. Mandelie van der Walt: formal analysis, writing – review and editing, visualisation. June C. Serem: resources. A. James Mason: writing – review and editing, funding acquisition. Megan J. Bester: conceptualisation, resources, writing – review and editing, supervision. Anabella R. M. Gaspar: conceptualisation, resources, writing – review and editing, supervision, project administration, funding acquisition.

## Conflicts of interest

There are no conflicts to declare.

## Supplementary Material

TB-014-D6TB00168H-s001

## Data Availability

Data for this article are available at Figshare at https://doi.org/10.6084/m9.figshare.31099765. Supplementary information (SI) is available. See DOI: https://doi.org/10.1039/d6tb00168h.
