# Supplementary material for: Tyrosine-capped gold nanoparticles enable cysteine-free peptide loading, enhancing the antipseudomonal efficacy of scorpion-derived AamAP1-Lys-NH2 in a burn wound infection model
Source: J Mater Chem B. 2026 Jun 22;14(27):8427–43. doi: 10.1039/d6tb00168h (PMC13284850; doi:10.1039/d6tb00168h)
Supplement: TB-014-D6TB00168H-s001 [file TB-014-D6TB00168H-s001.pdf]

Supplementary Information (SI) for Journal of Materials Chemistry B.
This journal is © The Royal Society of Chemistry 2026

Tyrosine-capped gold nanoparticles enable cysteine-free peptide loading, enhancing the antipseudomonal efficacy of scorpion-derived AamAP1-Lys-NH_2 in a burn wound infection model

Rosalind J. Van Wyk[a], Mandelie van der Walt[a], June C. Serem[b], A. James Mason[c*], Megan J. Bester[b] and Anabella R. M. Gaspar[a*]

[a] Department of Biochemistry, Genetics and Microbiology, Faculty of Natural and Agricultural Sciences, University of Pretoria, 0002, South Africa

[b] Department of Anatomy, Faculty of Health Sciences, University of Pretoria, 0002, South Africa

[c] Institute of Pharmaceutical Science, School of Cancer & Pharmaceutical Sciences, King's College London, SE1 9NH, United Kingdom

Corresponding Authors

*Prof. ARM Gaspar, Department of Biochemistry, Genetics and Microbiology, Faculty of Natural and Agricultural Sciences, University of Pretoria, anabella.gaspar@up.ac.za

*Prof. AJ Mason, Institute of Pharmaceutical Science, School of Cancer & Pharmaceutical Sciences, King's College London, SE1 9NH, United Kingdom , james.mason@kcl.ac.uk

SUPPLEMENTARY DATA

Table S1. Representative determination of the percentage and amount of peptide bound in the AamAP1-Lys-NH_2-AuNPs and D-AamAP1-Lys-NH_2-AuNPs when prepared at pH 10.0

Parameter	AamAP1-Lys-NH_2-AuNP	D-AamAP1-Lys-NH_2-AuNP
Absorbance (a.u) of 400 µg/mL peptide stock at 218nm	2.305	2.248
Absorbance (a.u) of pep-AuNP supernatant at 218nm	-0.048 (taken as 0.00)	-0.055 (taken as 0.00)
% peptide in supernatant	~0%	~0%
% binding of peptide to AuNP	~100%	~100%
Amount of peptide bound	400 µg	400 µg

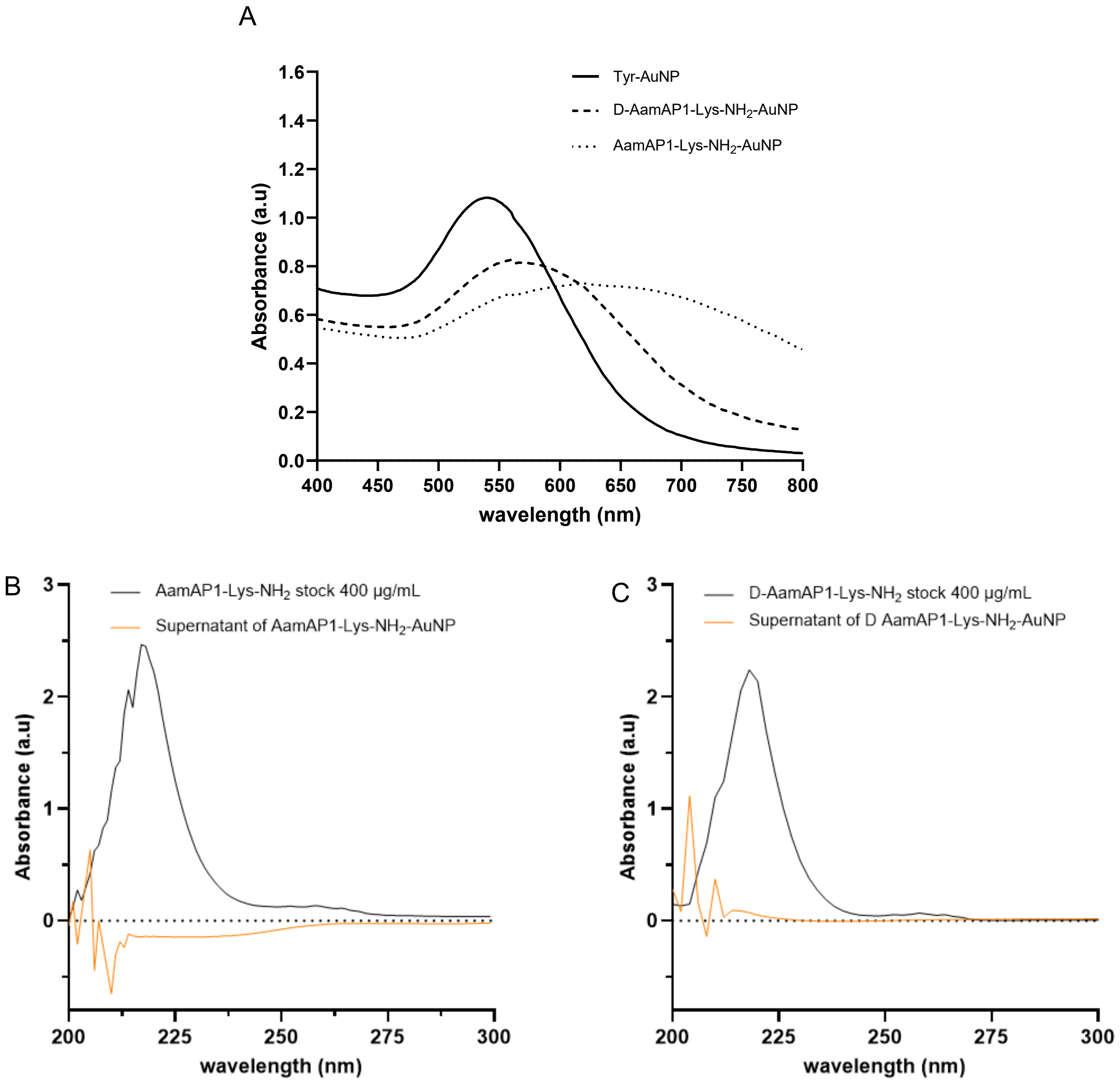

Figure S1. Synthesis of Tyr- and AMP-AuNPs and their characterization with UV-Vis spectroscopy. *A) Second representative UV-Vis spectra of Tyr-AuNP, AamAP1-Lys-NH_2-AuNP and D-AamAP1-Lys-NH_2-AuNP (100 µg/mL) showing the SPR band shift after addition to AMPs. B) Second representative UV-Vis spectra of the AamAP1-Lys-NH_2 stock solution (400 µg/mL) and the AamAP1-Lys-NH_2-AuNP supernatant, showing absence of peptide in the supernatant, suggesting successful conjugation to AuNPs and precipitation in the pellet. C) Second representative UV-Vis spectra of the D-AamAP1-Lys-NH_2 stock solution (400 µg/mL) and the D-AamAP1-Lys-NH_2-AuNP supernatant, showing absence of peptide in the supernatant, suggesting successful conjugation to AuNPs and precipitation in the pellet.*

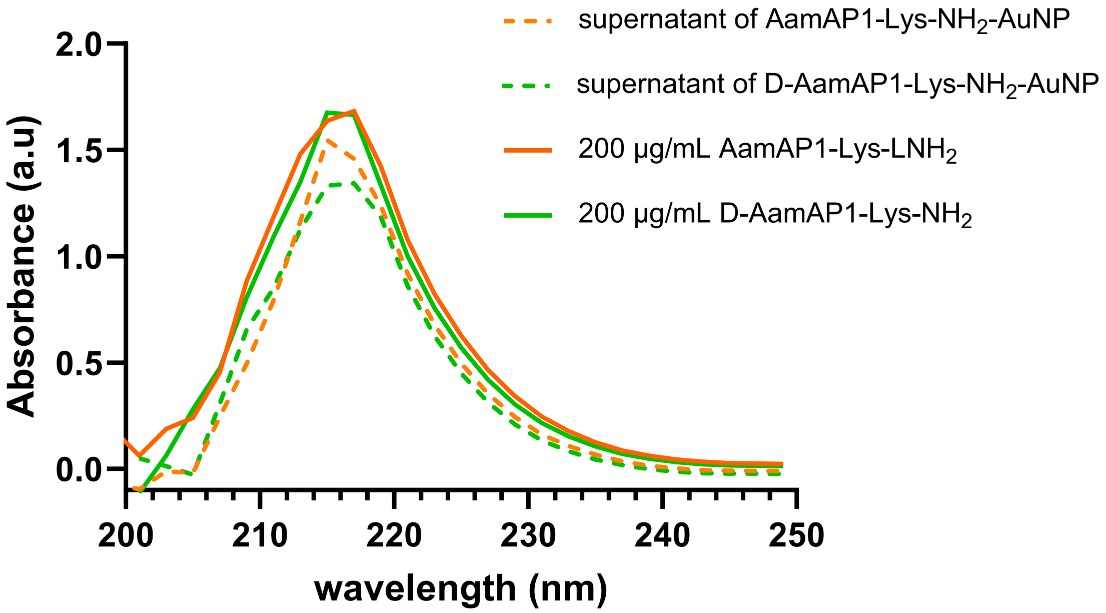

Figure S2. Representative UV-Vis spectra of AamAP1-Lys-NH_2-AuNP and D-AamAP1-Lys-NH_2-AuNP supernatants after preparation at pH 7.0 compared with 200 µg/mL of free AamAP1-Lys-NH_2 and D-AamAP1-Lys-NH_2 stock solutions. *If the pH is not adjusted to 10.0 after the addition of Tyr-AuNPs to the dry AMPs, peptide loading onto the nanoparticles is significantly reduced. As a result, less peptide is associated with the nanoparticle pellet and more remains in the supernatant with levels comparable to those observed with 200 µg/mL of free AMPs.*

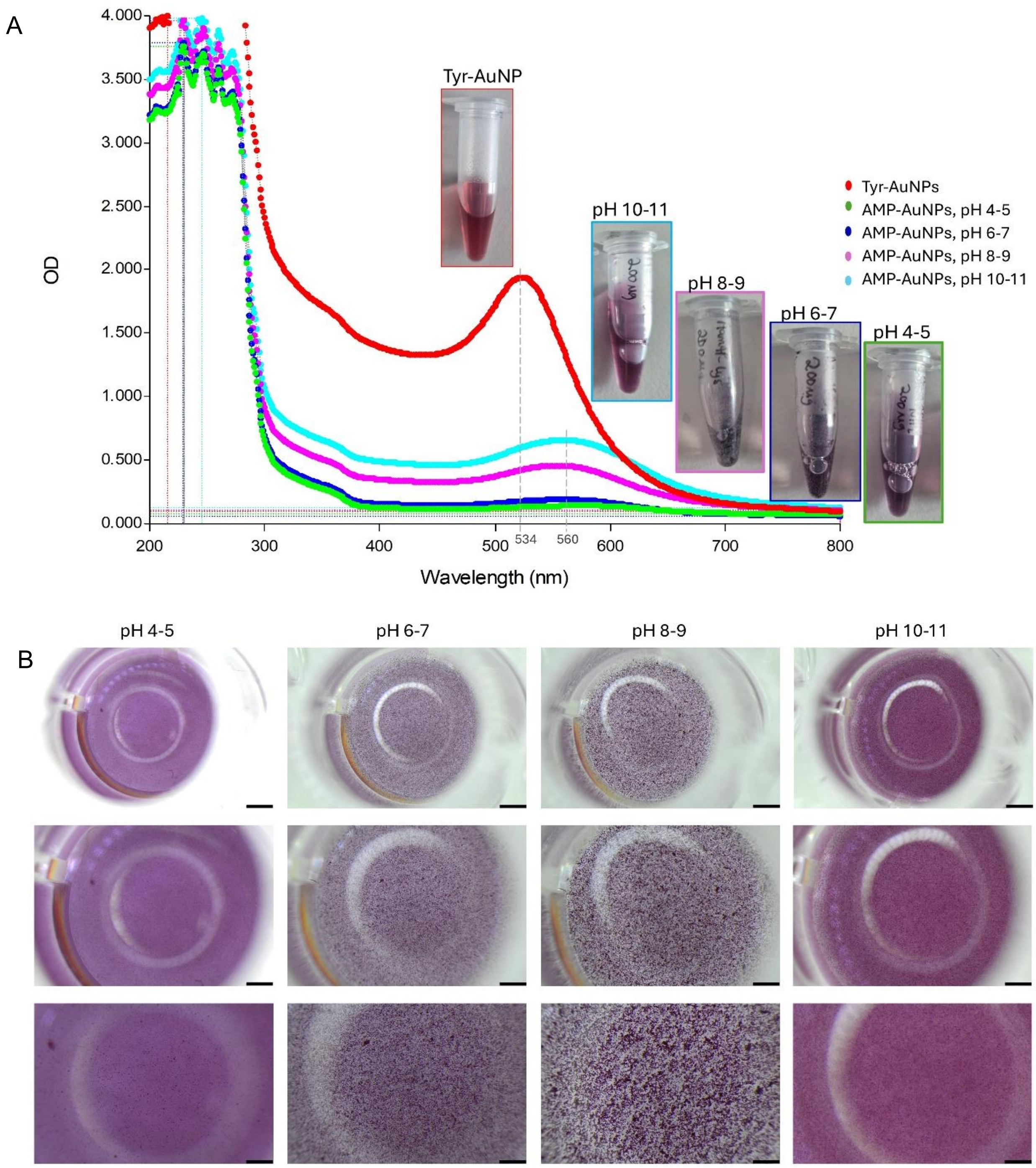

Figure S3. Aggregation of AMP-AuNPs synthesized at different pH values, characterized by UV-Vis spectroscopy and stereomicroscopy. *A) Visible colour changes of AuNP solutions before and after the addition of AMPs from ruby red (Tyr-AuNP) to purple hues depending on pH. Representative UV-Vis spectra of Tyr-AuNP and AamAP1-Lys-NH$_2$-AuNP showing SPR band red shifts and broadening following AMP addition across pH ranges 4-5, 6-7, 8-9 and 10-11. B) Stereomicroscopy images showing pH dependent aggregation of AMP-AuNPs at magnifications of 1.6x, 2.5x, and 4x. Scale bars = 2 mm.*

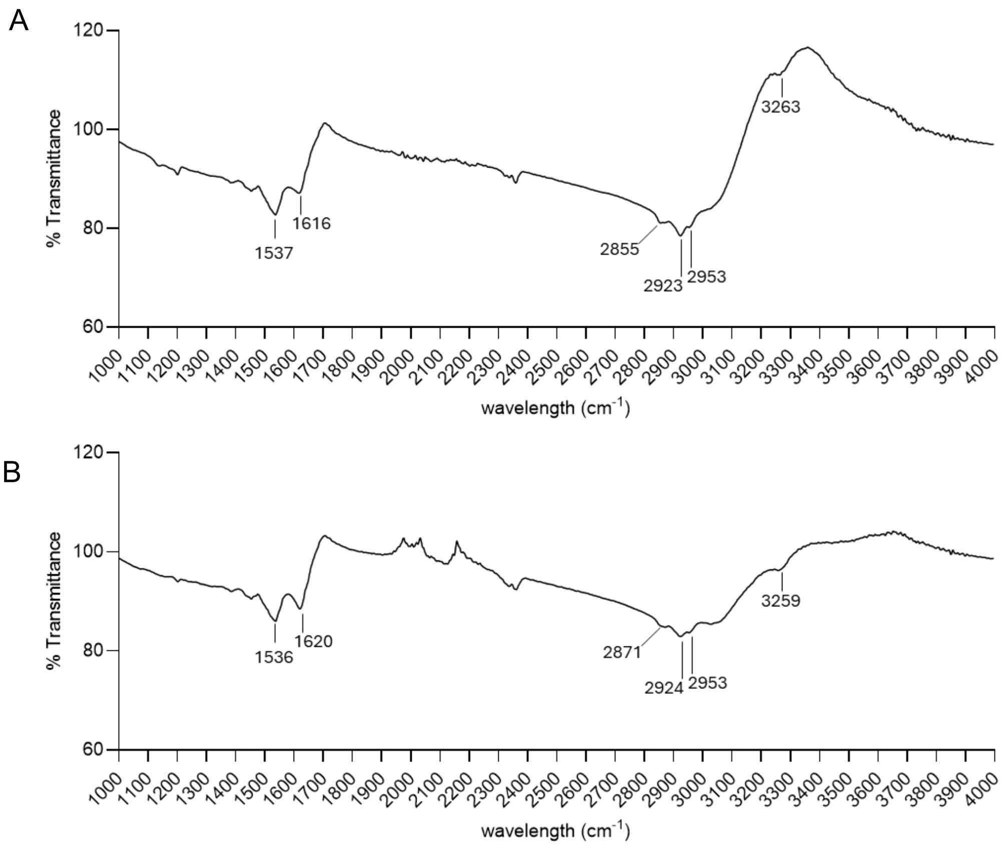

Figure S4. ATR-FTIR spectra of free A) AamAP1-Lys-NH$_2$ and B) D-AamAP1-Lys-NH$_2$ with 4 cm^{-1} resolution recorded between 1000 – 4000 cm^{-1}. *The main transmittance minima, corresponding to characteristic absorption bands of peptide backbone and side-chain functional groups, are labelled with their respective wavelengths. The presence of amide linkages is typically shown at 1540 ± 10 cm^{-1}, stretching of the benzene ring C=C bonds in the aromatic rings is shown at 1600 ± 5 cm^{-1}, methyl groups are shown at 2850-2880 cm^{-1}, C–H stretching bands at 2920 ± 10 cm^{-1}, terminal methyl groups (–CH$_3$) at 2950 ± 10 cm^{-1} and presence of peptide N–H and/or O–H groups at 3250-3270 cm^{-1}.*

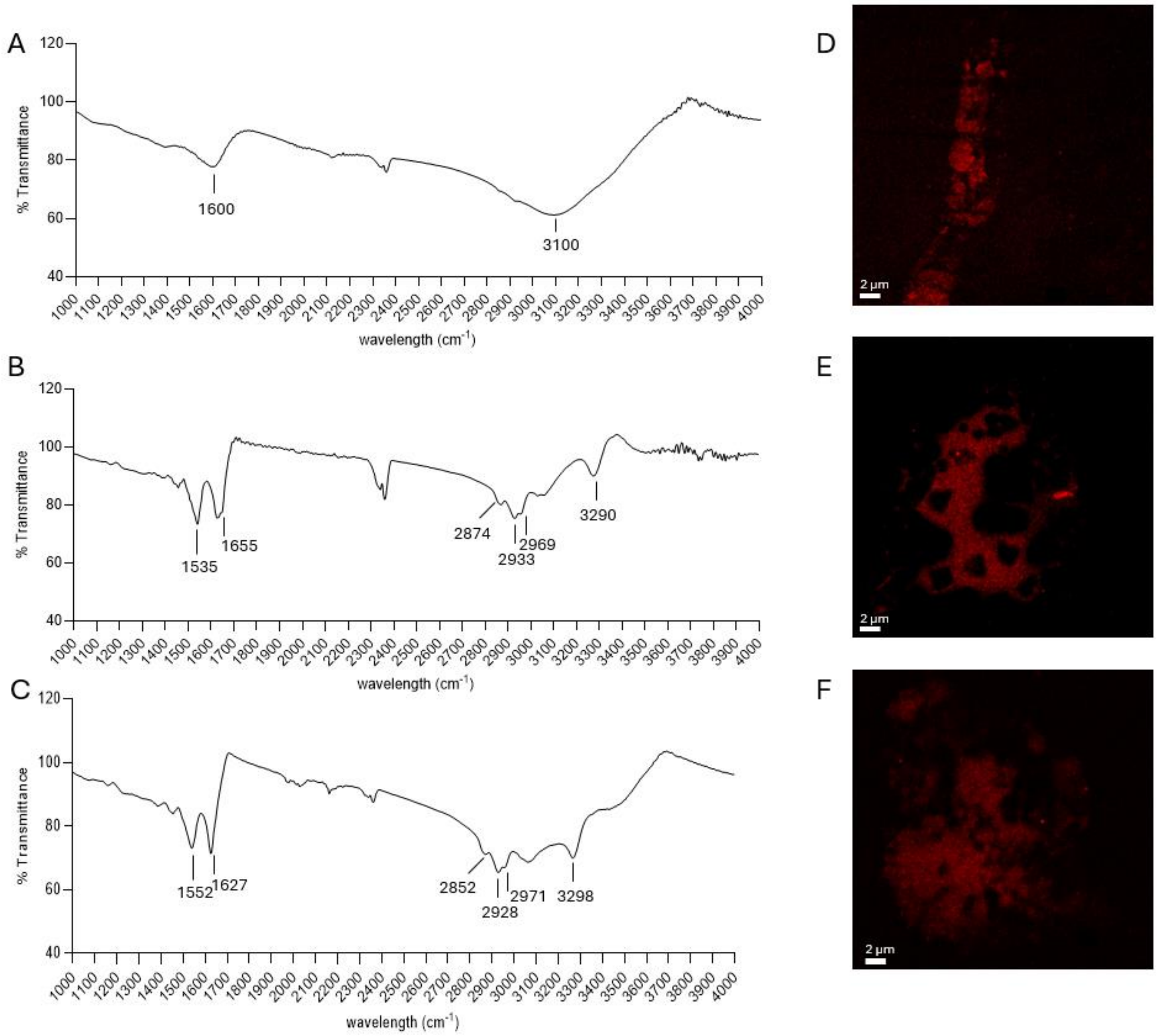

Figure S5. Second repeat of FTIR spectroscopy and confocal microscopy: Surface of AuNPs changes after loading of AMPs*. Characterization of the surface of the Tyr-AuNP and AMP-AuNPs using FTIR spectroscopy and confocal microscopy. Second representative of the ATR-FTIR spectra of A) Tyr-AuNPs, B) AamAP1-Lys-NH_2-AuNPs and C) D-AamAP1-Lys-NH_2-AuNPs with 4 cm^{-1} resolution recorded between 1000 – 4000 cm^{-1}. Second representative of duplicate confocal fluorescence images of D) Tyr-AuNPs, E) AamAP1-Lys-NH_2-AuNPs and F) D-AamAP1-Lys-NH_2-AuNPs excited at λ = 514 nm, shown with a scale = 2 µm.*

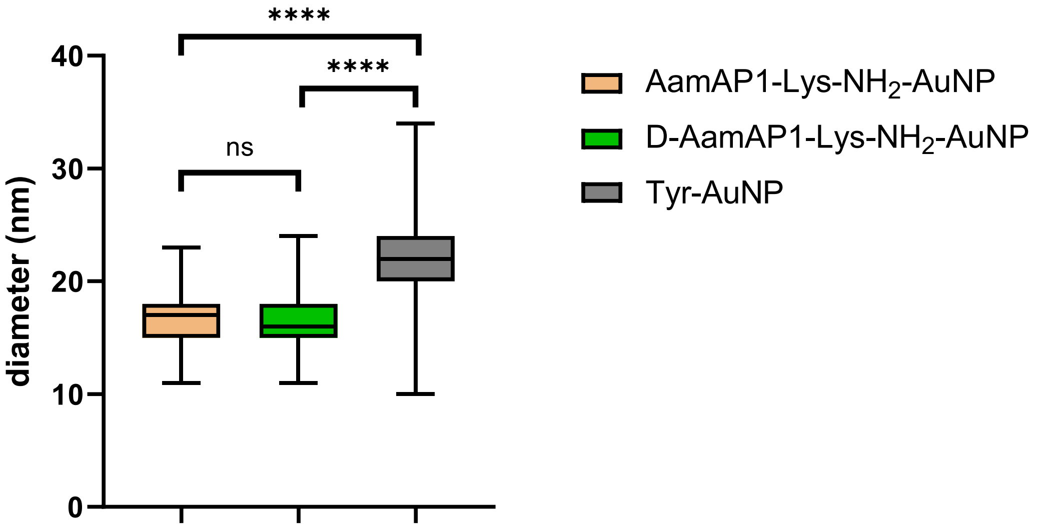

Figure S6. The average diameter of AamAP1-Lys-NH$_2$, D-AamAP1-Lys-NH$_2$ and Tyr-AuNP determined from TEM images. *A total of 200 measurements were taken for each AuNP, from two independent repeats. Statistical significance indicated by **** represent $p < 0.0001$ as determined from ordinary one-way ANOVA with Tukey's multiple comparison test.*

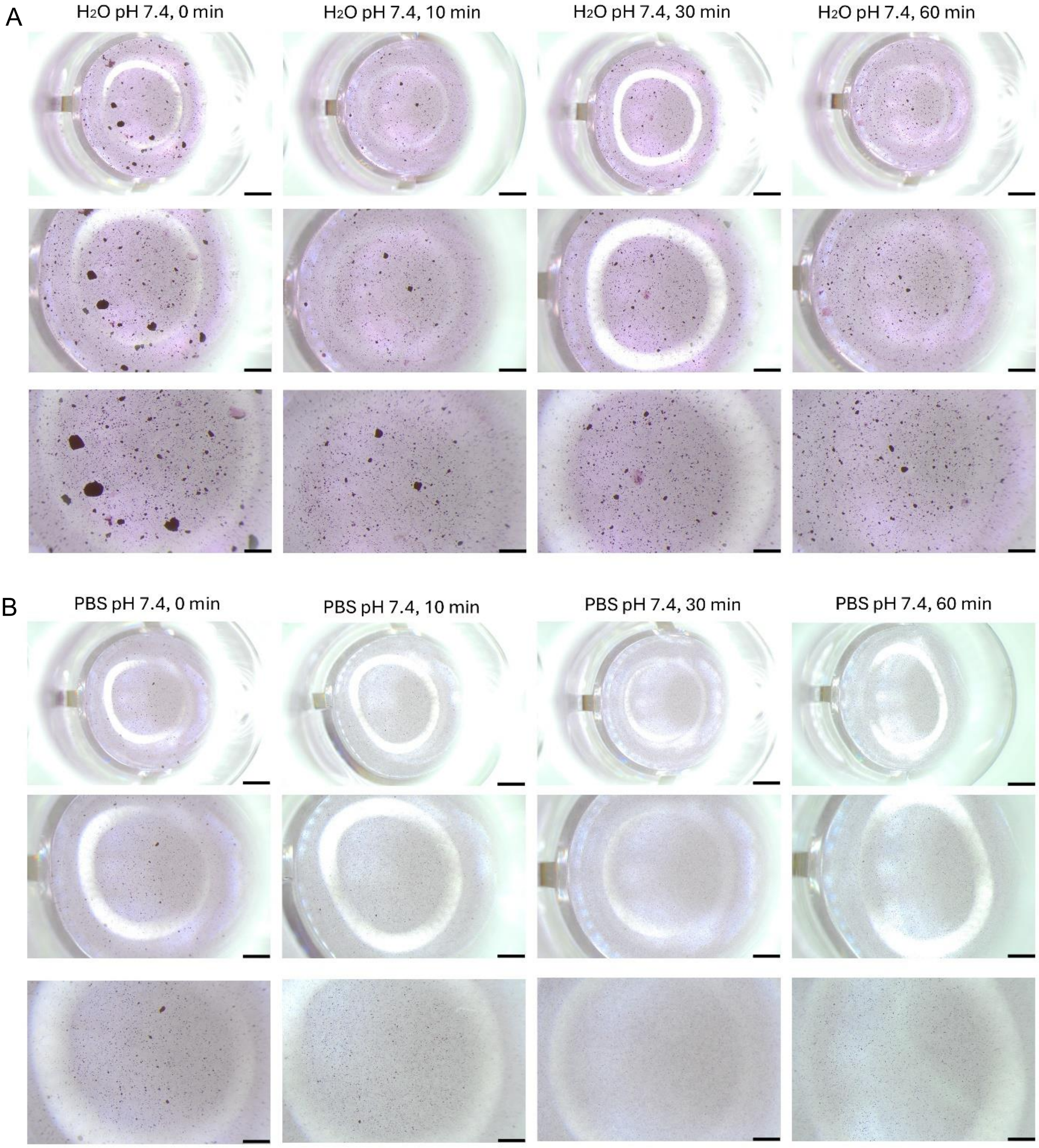

Figure S7. Dissociation of AMP-AuNP aggregates in water and PBS over time. *Stereomicroscopy images showing the time-dependent dissociation of AMP-AuNP aggregates at magnifications of 1.6x, 2.5x, and 4x in A) ddH$_2$O (pH 7.0) and B) PBS (pH 7.4). Scale bars = 2 mm.*
